# Traditional usages, chemical metabolites, pharmacological activities, and pharmacokinetics of *Boesenbergia rotunda* (L.) Mansf.: a comprehensive review

**DOI:** 10.3389/fphar.2025.1527210

**Published:** 2025-03-19

**Authors:** Yan Wang, Juanjuan Wen, Feng Liu, Xiujuan Peng, Gang Xu, Mingliang Zhang, Zhuangzhuang Huang

**Affiliations:** ^1^ Key Laboratory of Chemical Substances and Biological Effects in Traditional Chinese Medicine, College of Medicine and Pharmacy, Shaanxi Institute of International Trade & Commerce, Xi’an, Shaanxi, China; ^2^ Shaanxi Buchang Pharmaceutical Co. Ltd, Xi’an, Shaanxi, China; ^3^ Department of Pharmacy, The First Affiliated Hospital of Henan University of Chinese Medicine, Zhengzhou, Henan, China

**Keywords:** *Boesenbergia rotunda* (L.) Mansf, traditional usages, flavonoids, anti-cancer activity, quality control, toxicology, clinical settings

## Abstract

*Boesenbergia rotunda*: (L.) Mansf. (family Zingiberaceae), also known as fingerroot, is a medicinal and food plant that is widely distributed in southern China, Southeast Asia, and South Asia. It is a traditional herb and spice that is also known for its beneficial effects on *Qi*, appetite, stagnation and pain relief. The objective of this study is to conduct a comprehensive and systematic review of the botanical characteristics, traditional applications, phytochemical metabolites, pharmacological properties, toxicology, quality control measures, pharmacokinetics, and clinical applications of *B. rotunda*. A bibliometric analysis of current studies on *B. rotunda* was also conducted to facilitate further exploration and utilization of *B. rotunda* in the functional food and pharmaceutical industries. These data were collected from PubMed, Web of Science, Google Scholar, China National Knowledge Infrastructure doctoral and master’s theses and other books and scientific databases by searching the keywords *Boesenbergia rotunda*. Phytochemical analysis has revealed the presence of flavonoids, monoterpenes, alkaloids, aromatic metabolites, phenols, and other metabolites in *B. rotunda*, exhibiting a wide range of biological activities such as anti-cancer, nephroprotective, anti-inflammatory, anti-bacterial, hepatoprotective, anti-obesity, and anti-oxidant effects, both *in vivo* and *in vitro*. In this paper, the research of *B. rotunda* is discussed in depth by combining traditional application and modern pharmacological research, aiming to provide valuable reference for the future research and practical application of *B. rotunda*.

## Graphical Abstract

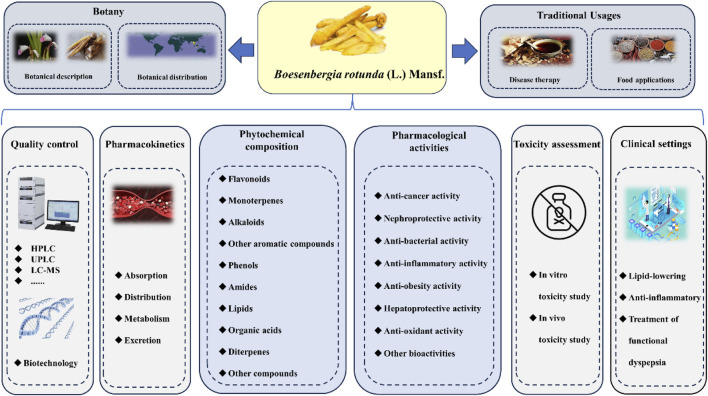

## 1 Introduction

**FIGURE 1:**
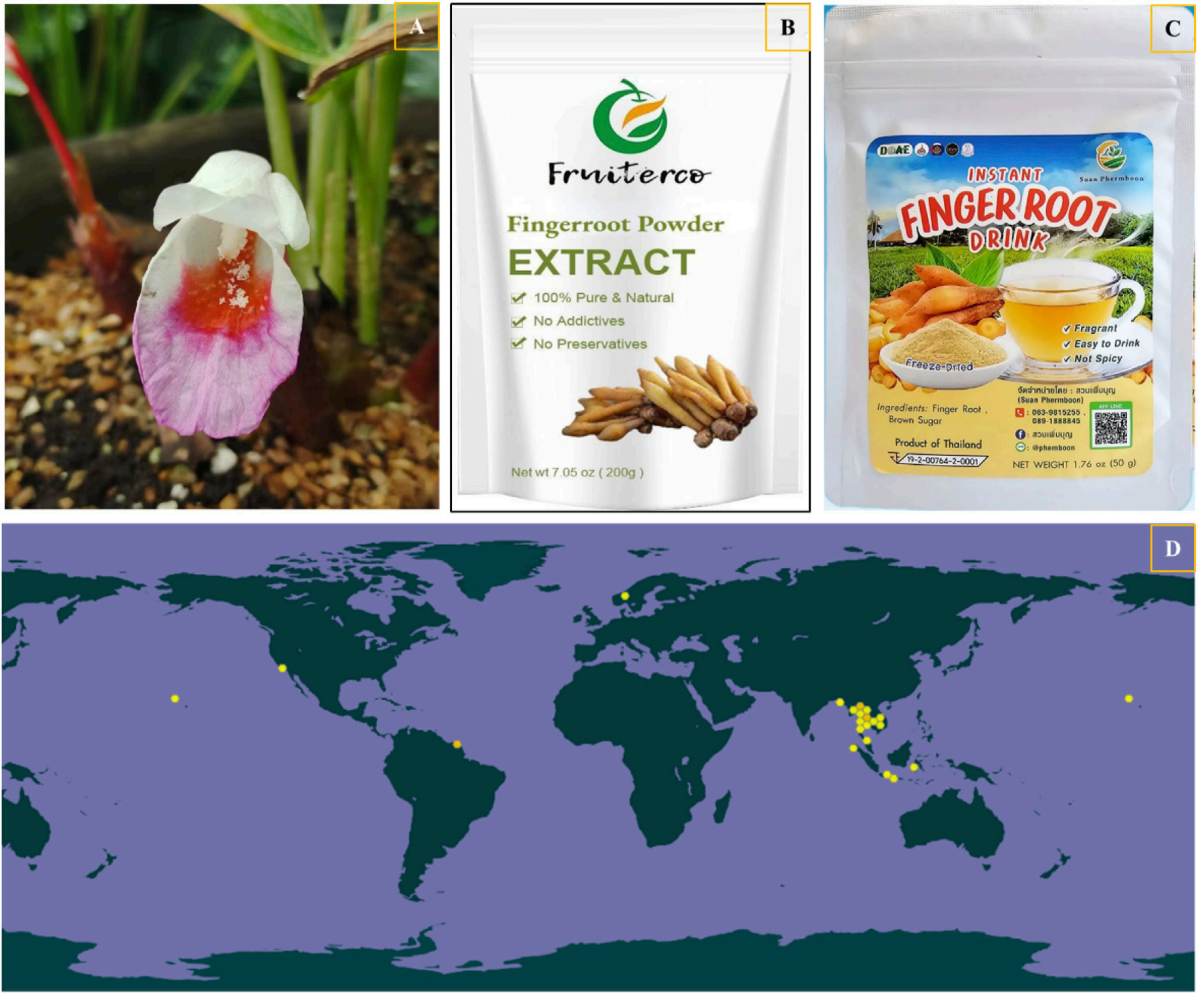
The ornamental value of *B. rotunda* **(A)**, products **(B, C)**, along with a global distribution map of *B. rotunda* **(D)**, can be accessed at the global biodiversity Information Facility (GBIF) website (https://www.gbif.org/species/2758480).

*Boesenbergia rotunda* (L.) Mansf., belonging to the Zingiberaceae family and colloquially referred to as fingerroot, is a versatile plant with both medicinal and culinary applications. It is extensively integrated into regional gastronomy and traditional medicinal practices. This plant is renowned for its diverse health benefits and is indigenous to Southern China, Southeast Asia, and South Asia (Vergoten and Bailly, 2022; Kaewjai et al., 2023). It has high ornamental value, medicinal value and edible value (Figures 1A–C) (Apiratikul et al., 2023; Zhang et al., 2023). *B. rotunda* is predominantly distributed across India, Myanmar, Thailand, China, Malaysia, and so on. In the pharmacopoeias of Vietnam, Cambodia, Laos and China, the rhizomes of this species have traditionally been recognized for their therapeutic effects, including reducing flatulence, relieving fatigue, improving symptoms of menstrual cramps, and boosting bile production (Figure 1D) (Kongratanapasert et al., 2023). The leaves of *B. rotunda* are notably broad and bear a resemblance to those of the banana plant, complemented by the plant’s aesthetically pleasing flowers. These characteristics give it some ornamental value (Jamornwan et al., 2022). In the field of medicine, the rhizome of *B. rotunda* is recognized as a medicinal botanical with distinctive pungent characteristics and warm thermogenic properties in traditional Chinese medicine. It is particularly esteemed for its multifaceted therapeutic potential, which includes the facilitation of *Qi* circulation, enhancement of appetite, mitigation of stagnation, and alleviation of pain. These pharmacological actions render *B. rotunda* an effective agent in the management of gastrointestinal distension, diarrhea, and an array of associated gastrointestinal disorders (Nguyen et al., 2021). At present, because of its rich medicinal value, it has been widely used in clinical treatment (Kongwattanakul et al., 2022). In Southeast Asia and other places, *B. rotunda* is still a commonly used seasoning for fish, vegetables and curry seasoning, which can remove the smell of food, and has a variety of effects such as nourishing (Rungsa et al., 2023).

In recent years, *B. rotunda* has received increasing attention for its profound medicinal and nutritional value. Many scholars have conducted extensive and far-reaching research on *B. rotunda* (Techapichetvanich et al., 2024). Currently, over 200 distinctive metabolites have been isolated and identified through phytochemical investigations of various parts of *B. rotunda*. These include flavonoids, monoterpenes, alkaloids, aromatics, phenols, amides, esters, organic acids, diterpenes, and other metabolites. Notably, the abundant flavonoids and monoterpenes impart a unique fragrance to *B. rotunda*, enhancing its clinical application potential. Contemporary pharmacological studies reveal that the extracts and isolated metabolites exhibit a diverse range of biological activities, including significant anti-cancer activity (Widyananda et al., 2022), nephroprotective activity (Sujana et al., 2024), anti-inflammatory activity (Liana et al., 2024), anti-bacterial activity (Thadtapong et al., 2024), anti-obesity activity (Rungsa et al., 2023), hepatoprotective activity (Salama et al., 2018), anti-oxidant activity (Saah et al., 2024), and other bioactivities (Kaewjai et al., 2023). Furthermore, the rich nutritional profile and salutary metabolites present in *B. rotunda* offer considerable promise for application within the sphere of natural animal nutrition supplements and feed additives (Yushinta et al., 2023).

Despite the large number of research results on *B. rotunda*, the existing literature still lacks a holistic and systematic overview of *B. rotunda*, and the understanding of *B. rotunda* lacks a certain degree of comprehensiveness. The aim of this review is to improve the existing research on *B. rotunda* and critically summarize the latest progress, including geographical distribution, botanical characteristics, ethnomedical applications, phytochemical metabolites, pharmacological activities, quality control, toxicity assessment, and clinical settings. The ultimate goal of this review is to establish a solid scientific foundation for continued research, development, and application of *B. rotunda*, as well as to indicate potential avenues for future exploration and innovation.

## 2 Methodology

A systematic literature search was performed utilizing online databases including PubMed, Elsevier, Web of Science, and China National Knowledge Infrastructure (CNKI), as well as repositories of doctoral and master’s theses. The search employed a variety of keywords, such as “*B. rotunda*”, “fingerroot”, and “*B. rotunda* (L.) Mansf”, to compile an extensive corpus of relevant literature. The data, encompassing aspects of botany, ethnopharmacology, phytochemistry, pharmacology, and toxicology pertaining to *B. rotunda*, were meticulously synthesized. Chemical metabolites identified were delineated using ChemDraw software. And bibliometric analysis of *B. rotunda* using VOSviewer, Web of Science, etc. The literature review spanned an unrestricted time frame, with eligible publications predominantly dating from 2002 to 2024.

## 3 Botanical descriptions

**FIGURE 2:**
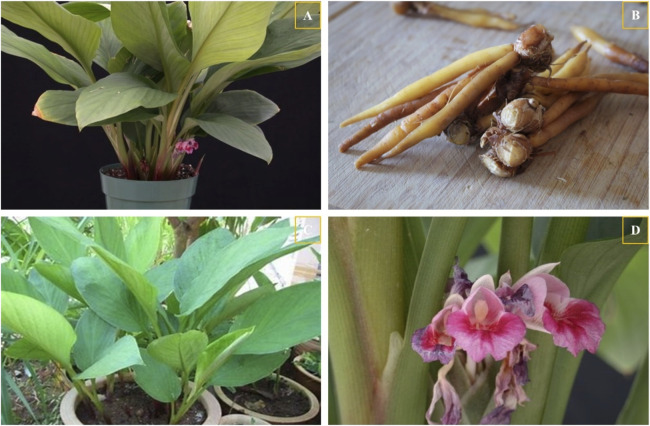
The whole plant **(A)**, roots **(B)**, stems and leaves **(C)**, and flowers **(D)** of *B. rotunda* (https://www.gbif.org/species/2758480).

*B. rotunda* is a perennial herbaceous species, attaining a stature of approximately 50 cm, and is notable for the absence of a conspicuous aerial stem (Figure 2A). The subterranean rhizome of this plant is ovate, yellowish in color, possesses a distinctive aroma, and exhibits a substantial thickness (Figure 2B) (Taheri et al., 2022). The foliar blades of *B. rotunda*, situated at the plant’s base, display an ovate-elongated to elliptic-lanceolate morphology, with lengths spanning from 25 to 50 cm and widths ranging from 7 to 12 cm. These leaves are distinguished by an acutely pointed apex and a base that tapers to a broadly rounded form (Figure 2C) (Bahadur Gurung et al., 2022). The inflorescence of *B. rotunda*, which resembles a spike, emerges exclusively from the rhizome and comprises three to six flowers (Figure 2D). The bracts are ovate, measuring 2–4 cm in length, with an acuminate apex and exhibit a pubescent texture. The flowers exhibit an enchanting blue-purple coloration. The calyx, 1–2 cm in length, features a cleft on one of its sides. The corolla tube can extend up to a maximum length of 4 cm, adorned with lanceolate lobes that measure 2.2–2.5 cm in length. The lateral staminodes are congruent in length to the corolla lobes and are oval in shape. The lip flap, which is obovate and cuneate, measures approximately 3 cm in length. The filaments are notably short, while the anthers are broad, characterized by linear appendages and linear anther locules, which are approximately 1 cm in length and bear a thickened stigma (https://www.worldfloraonline.org/taxon/wfo-0000341663). *B. rotunda* exhibits a distinctive growth pattern, flourishing in warm, humid, and semi-shaded environments. It is optimally suited for cultivation within tropical monsoon forests at elevations ranging from 700 to 1,900 m. The distribution of *B. rotunda* spans across the tropical monsoon forests of Southeast Asia, including regions in Malaysia, Singapore, Thailand, and the Philippines, as well as extending to the South Asian subcontinent, notably India and Sri Lanka, with sporadic occurrences in other locales (Liew et al., 2022). Additionally, *B. rotunda* is also found in Yunnan and other regions within China, where it has become a traditional Chinese medicine (Zhang et al., 2023).

## 4 Traditional usages

*B. rotunda* is native to the Indonesian archipelago, specifically the islands of Java or Sumatra (Vergoten and Bailly, 2022). The plant is known in Southeast Asia for its healing properties. It is employed in the treatment of a spectrum of conditions, encompassing fever, rheumatism, muscular discomfort, peptic ulcers, gastrointestinal disturbances, bacterial infections, and a variety of other health complaints (Yuliana et al., 2013; Wang et al., 2022; Taechowisa et al., 2024). The remarkable therapeutic efficacy of *B. rotunda* has catalyzed its extensive utilization throughout the Southeast Asian region. This is further substantiated by its recognition and inclusion in the monograph on Association of Southeast Asian Nations herbal standards, which serves as a testament to its established medicinal value in the region (Kanchanapiboon et al., 2020). In Indonesia, *B. rotunda* is highly regarded as a health-promoting medicinal resource, known for addressing male sexual dysfunction and female infertility (Potipiranun et al., 2018). Within the traditional Indonesian healthcare framework, *B. rotunda* is considered invaluable for *postpartum* recovery, earning it the colloquial title of “ginseng in ginger” (Md-Mustafa et al., 2014). In Thailand, the plant is known locally as Thai Ginseng, and its rhizome extract is used to enhance wound repair, create anti-aging skincare formulations, and also to relieve stomach discomfort, manage leukoplakia, treat abscesses, and address leucorrhea (Kanchanapiboon et al., 2020). Meanwhile, in the context of traditional Thai medicine, the rhizome of Zingiber officinale is frequently prescribed for gastrointestinal complaints, including bloating, dyspepsia, and peptic ulcers (Mohan et al., 2020). Furthermore, in South Korea, a daily intake of up to 700 mg of a standardized finger root extract has been deemed both safe and efficacious (Vergoten and Bailly, 2022). It is also significant to note that both the extract derived from *B. rotunda* and its principal active metabolite, panduratin A, have demonstrated anti-viral activities against SARS-CoV-2 (Kaewjai et al., 2023).

Furthermore, *B. rotunda* is a staple in Southeast Asian culinary practices, prized for its nuanced flavor profile and invigorating aroma (Kabir et al., 2020). In Cambodia, *B. rotunda* is traditionally thinly sliced and pickled with fish broth to prepare the iconic Khmer dish, ‘Khmer Rice Noodle Soup’ Vietnamese cuisine employs *B. rotunda* as both a metabolic enhancer and an aromatic agent in the renowned ‘Lemon Leaf Vegetable Stew’ Indonesian gastronomy incorporates the rhizomes to lend a unique flavor to various porridges (San et al., 2022). In Thailand, *B. rotunda* is a pivotal condiment in the cherished shrimp soup, a dish particularly favored by nursing mothers due to its reputed ability to significantly enhance lactation (Rungsa et al., 2023). Collectively, *B. rotunda* functions not only as a savory seasoning but also as a nutraceutical food, enriching culinary creations with its distinctive essence while concurrently providing potential health benefits through dietary intervention.

Future research should further explore the traditional applications of *B. rotunda*, explore the mechanism of action of *B. rotunda*, search for the best therapeutic program, establish comprehensive safety information, and comprehensively safeguard the traditional applications of *B. rotunda*. In addition, comprehensive research on its dual role of medicinal and edible uses will further explore its application in medicine, thus expanding its role in modern medicine. Explore its potential in the field of functional foods and develop effective and reliable health foods.

## 5 Phytochemical composition

**FIGURE 3:**
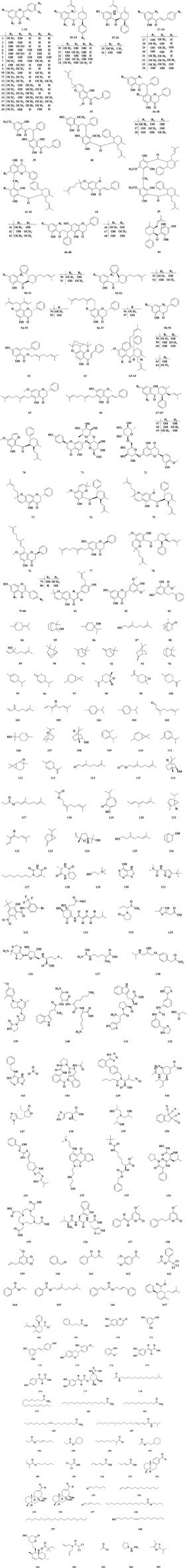
The structural compositions of the metabolites derived from *B. rotunda* have been elucidated.

To date, a total of approximately 205 chemical metabolites have been effectively extracted and characterized from diverse anatomical segments of *B. rotunda*. These metabolites mainly included flavonoids (**1–83**), monoterpenes (**84–126**), alkaloids (**127–156**), other aromatic metabolites (**157–169**), phenols (**170–177**), amides (**178–183**), lipids (**184–188**), organic acids (**189–191**), diterpenes (**192–194**), and other metabolites (**195–205**). Here, the present paper comprehensively compiled the chemical metabolites of bioactive metabolites and their related literature and chemical structures, as shown in Table 1 and Figure 3. This also encompasses the identification of new metabolites in recent years, which provides a foundation for the development of clinical drugs.

**TABLE 1:** Chemical metabolites isolated and identified from the different parts of *Boesenbergia rotunda* (L.) Mansf.

No.	Chemical metabolites	Molecular formula	Extracts	Parts	Refs
Flavonoids					
1	Pinostrobin	C_16_H_14_O_4_	CO_2_ SFE	Rhizomes	Techapichetvanich et al. (2024)
			EtOH	Rhizomes	Thongrong et al. (2023)
			Aqueous	Rhizomes	Liana et al. (2024)
			Hexane	Rhizomes	Liana et al. (2024)
			DCM	Rhizomes	Kabir et al. (2020)
			CHCl_3_	Rhizomes	Win et al. (2019)
			MeOH	Roots	Chatsumpun et al. (2017)
2	Pinocembrin	C_15_H_12_O_4_	MeOH	Roots	Rungsa et al. (2023)
			EtOH	Rhizomes	Chuah et al. (2022)
			DCM	Rhizomes	Kabir et al. (2020)
			CHCl_3_	Rhizomes	Tuchindaa et al. (2002)
3	Alpinetin	C_16_H_14_O_4_	MeOH	Roots	Rungsa et al. (2023)
			DCM	Rhizomes	Kabir et al. (2020)
4	Kaempferol	C_15_H_10_O_6_	EtOH	Rhizomes	Ruttanapattanakul et al. (2021)
			MeOH	Rhizomes	Ling et al. (2010)
5	Naringenin 5-methyl ether	C_16_H_14_O_5_	MeOH	Rhizomes	Adhikari et al. (2020)
6	Quercetin	C_15_H_10_O_7_	EtOH	Rhizomes	Rosdianto et al. (2020)
			MeOH	Rhizomes	Ling et al. (2010)
7	4′,7-dimethylkaempferol	C_17_H_14_O_6_	CHCl_3_	Rhizomes	Win et al. (2019)
8	7,4′-Dihydroxy-5-methoxyflavanone	C_16_H_14_O_5_	MeOH	Roots	Chatsumpun et al. (2017)
			EtOH	Rhizomes	Win et al. (2007)
9	5,7-Dimethoxyflavone	C_17_H_14_O_4_	Hexane	Rhizomes	Jaipetch et al. (1982)
10	5-Hydroxy-7,4′-dimethoxyflavon	C_17_H_14_O_5_	Hexane	Rhizomes	Jaipetch et al. (1982)
11	5,7,4′-Trimethoxyflavone	C_18_H_16_O_5_	Hexane	Rhizomes	Jaipetch et al. (1982)
12	5,7,3′,4′-Tetramethoxyflavone	C_19_H_18_O_6_	Hexane	Rhizomes	Jaipetch et al. (1982)
13	5-Hydroxy-3,7-dimethoxyflavone	C_17_H_14_O_5_	Hexane	Rhizomes	Jaipetch et al. (1982)
14	5-Hydroxy-3,7,4′-trimethoxyflavone	C_18_H_16_O_6_	Hexane	Rhizomes	Jaipetch et al. (1982)
15	3,5,7-Trimethoxyflavone	C_18_H_16_O_5_	Hexane	Rhizomes	Jaipetch et al. (1982)
16	5-Hydroxy-3,7,3′,4′-tetramethoxyflavone	C_19_H_18_O_7_	Hexane	Rhizomes	Jaipetch et al. (1982)
17	3,5,7,3′,4′-Pentamethoxyflavone	C_20_H_20_O_7_	Hexane	Rhizomes	Herunsalee et al. (1987)
18	3,5,7,4′-Tetramethoxyflavone	C_19_H_18_O_6_	Hexane	Rhizomes	Herunsalee et al. (1987)
19	Sakuranetin	C_16_H_14_O_5_	EtOH	Rhizomes	Chuah et al. (2022)
20	Panduratin A	C_26_H_30_O_4_	CO_2_ SFE	Rhizomes	Techapichetvanich et al. (2024)
			EtOH	Rhizomes	Thadtapong et al. (2024)
			DCM	Rhizomes	Kabir et al. (2020)
			MeOH	Roots	Chatsumpun et al. (2017)
			CHCl_3_	Rhizomes	Tuchindaa et al. (2002)
21	(−)-Isopanduratin A	C_26_H_30_O_4_	MeOH	Roots	Rungsa et al. (2023)
			DCM	Rhizomes	Kabir et al. (2020)
22	(−)-4-Hydroxypanduratin A	C_25_H_28_O_4_	MeOH	Rhizomes	Adhikari et al. (2020)
23	Panduratin C	C_26_H_30_O_5_	MeOH	Rhizomes	Cheenpracha et al. (2006)
			EtOH	Rhizomes	Win et al. (2007)
24	(±)-6-Methoxypanduratin A	C_27_H_32_O_5_	EtOH	Rhizomes	Win et al. (2007)
25	(+)-Isopanduratin A	C_26_H_30_O_4_	MeOH	Rhizomes	Morikawa et al. (2008)
26	(+)-4-Hydroxypanduratin A	C_25_H_28_O_4_	MeOH	Rhizomes	Morikawa et al. (2008)
			MeOH	Roots	Rungsa et al. (2023)
			CHCl_3_	Rhizomes	Tuchindaa et al. (2002)
27	Cardamonin	C_16_H_14_O_4_	MeOH	Roots	Rungsa et al. (2023)
			DCM	Rhizomes	Kabir et al. (2020)
28	Pinostrobin chalcone	C_16_H_14_O_4_	MeOH	Rhizomes	Adhikari et al. (2020)
29	Helichrysetin	C_16_H_14_O_5_	MeOH	Roots	Chatsumpun et al. (2017)
30	Pinocembrin chalcone	C_15_H_12_O_4_	MeOH	Stems	Tan et al. (2015)
31	2′-Hydroxy-4′,6′-dimethoxychalcone	C_17_H_16_O_4_	Hexane	Rhizomes	Herunsalee et al. (1987)
32	2′-Hydroxy-4,4′,6′-trimethoxychalcone	C_18_H_20_O_5_	Hexane	Rhizomes	Herunsalee et al. (1987)
33	Flavokawain C	C_17_H_16_O_5_	EtOH	Rhizomes	Win et al. (2007)
34	Naringenin chalcone	C_15_H_12_O_5_	EtOH	Rhizomes	Chuah et al. (2022)
35	Boesenbergin A	C_26_H_28_O_4_	EtOH	Rhizomes	Wang et al. (2022)
			Hexane	Rhizomes	Isa et al. (2012)
36	Panduratin Q	C_31_H_26_O_8_	MeOH	Rhizomes	Nguyen et al. (2021)
37	Panduratin R	C_31_H_26_O_8_	MeOH	Rhizomes	Nguyen et al. (2021)
38	Panduratin S	C_31_H_26_O_8_	MeOH	Rhizomes	Nguyen et al. (2021)
39	Panduratin T	C_31_H_28_O_8_	MeOH	Rhizomes	Nguyen et al. (2021)
40	Panduratin U	C_42_H_44_O_8_	MeOH	Rhizomes	Nguyen et al. (2021)
41	Panduratin V	C_42_H_42_O_8_	MeOH	Rhizomes	Nguyen et al. (2021)
42	Panduratin W	C_42_H_42_O_8_	MeOH	Rhizomes	Nguyen et al. (2021)
43	Panduratin X	C_43_H_44_O_8_	MeOH	Rhizomes	Nguyen et al. (2021)
44	Boesenbergin B	C_26_H_28_O_4_	DCM	Rhizomes	Kabir et al. (2020)
45	Panduratin Y	C_53_H_60_O_8_	MeOH	Rhizomes	Nguyen et al. (2021)
46	Rotundaflavanochalcone	C_31_H_26_O_8_	MeOH	Roots	Rungsa et al. (2023)
47	*iso*-rotundaflavanochalcone	C_31_H_26_O_8_	MeOH	Roots	Chatsumpun et al. (2017)
48	de-O-methyl rotundaflavanochalcone	C_30_H_24_O_8_	MeOH	Roots	Chatsumpun et al. (2017)
49	Hydroxy-panduratin	C_25_H_28_O_4_	Hexane	Rhizomes	Yuliana et al. (2013)
50	(+)-krachaizin A	C_26_H_30_O_4_	MeOH	Rhizomes	Morikawa et al. (2008)
51	(+)-krachaizin B	C_26_H_30_O_4_	MeOH	Rhizomes	Morikawa et al. (2008)
52	(−)-krachaizin A	C_26_H_30_O_4_	MeOH	Rhizomes	Morikawa et al. (2008)
53	(−)-krachaizin B	C_26_H_30_O_4_	MeOH	Rhizomes	Morikawa et al. (2008)
54	Rotundaflavones I	C_26_H_30_O_4_	MeOH	Rhizomes	Morikawa et al. (2008)
55	Rotundaflavones II	C_25_H_28_O_4_	MeOH	Rhizomes	Morikawa et al. (2008)
56	5,7-Dihydroxy-8-geranylflavanone	C_26_H_30_O_4_	MeOH	Rhizomes	Morikawa et al. (2008)
57	7-Methoxy-5-hydroxy-8-geranylflavanone	C_25_H_28_O_4_	MeOH	Rhizomes	Morikawa et al. (2008)
58	2,6-Dihydroxy-4-methoxydihydrochalcone	C_16_H_16_O_4_	MeOH	Rhizomes	Morikawa et al. (2008)
59	Uvangoletin	C_16_H_16_O_4_	MeOH	Rhizomes	Cheenpracha et al. (2006)
60	2′,4′,6′-Trihydroxydihydrochalcone	C_15_H_14_O_4_	MeOH	Roots	Rungsa et al. (2023)
61	Geranyl-2,4-dihydroxy-6-phenylbenzoate	C_25_H_30_O_4_	MeOH	Rhizomes	Morikawa et al. (2008)
			EtOH	Rhizomes	Win et al. (2007)
62	Rubranine	C_25_H_26_O_4_	Hexane	Rhizomes	Tuntiwachwuttik et al. (1984)
63	(±)-Panduratin B1	C_37_H_46_O_3_	Hexane	Rhizomes	Acharoen et al. (1987)
64	(±)-Panduratin B2	C_38_H_48_O_3_	Hexane	Rhizomes	Acharoen et al. (1987)
65	(2S)-6-Geranylpinostrobin	C_26_H_30_O_4_	EtOH	Rhizomes	Cheenpracha et al. (2006)
66	2′,4′-Dihydroxy-3'-(1″-geranyl)-6′-methoxychalcone	C_26_H_30_O_4_	EtOH	Rhizomes	Win et al. (2007)
67	(1′R,2′S,6′R)-2-Hydroxyisopanduratin A	C_25_H_28_O_4_	EtOH	Rhizomes	Win et al. (2007)
68	(±)-Isopanduratin A1	C_26_H_30_O_4_	EtOH	Rhizomes	Win et al. (2007)
69	Nicolaioidesin B	C_26_H_30_O_4_	EtOH	Rhizomes	Win et al. (2007)
70	Panduratin D	C_28_H_30_O_4_	EtOH	Rhizomes	Win et al. (2008)
71	Naringin	C_27_H_32_O_14_	MeOH	Rhizomes	Ling et al. (2010)
72	Hesperidin	C_28_H_34_O_15_	MeOH	Rhizomes	Ling et al. (2010)
73	(2S)-7,8-Dihydro-5-hydroxy-2-methyl-2-(4″-methyl-3″-pentenyl)-8-phenyl-2H,6H-benzo [1,2-b:5,4-b']dipyran-6-one	C_25_H_26_O_4_	EtOH	Rhizomes	Win et al. (2007)
74	Panduratin E	C_31_H_36_O_4_	EtOH	Rhizomes	Win et al. (2008)
75	Panduratin F	C_36_H_44_O_4_	EtOH	Rhizomes	Win et al. (2008)
76	(2R)-8-Geranylpinostrobin	C_26_H_30_O_4_	EtOH	Rhizomes	Win et al. (2007)
77	(−)-6-Geranylpinocembrin	C_25_H_28_O_4_	EtOH	Rhizomes	Win et al. (2007)
78	Panduratin G	C_36_H_44_O_4_	EtOH	Rhizomes	Win et al. (2008)
79	Biochanin A	C_16_H_12_O_5_	EtOH	Rhizomes	Chuah et al. (2022)
80	Chrysin	C_15_H_10_O_4_	EtOH	Rhizomes	Chuah et al. (2022)
81	Formononetin	C_16_H_12_O_4_	EtOH	Rhizomes	Chuah et al. (2022)
82	Paratocarpin B	C_25_H_26_O_4_	EtOH	Rhizomes	Chuah et al. (2022)
83	2,4-Dihydroxy-6-phenethylbenzoic acid methyl ester	C_16_H_16_O_4_	MeOH	Rhizomes	Morikawa et al. (2008)
Monoterpenoids					
84	Terpinolene	C_10_H_16_	Aqueous	Rhizomes	Apinundecha et al. (2023)
85	Camphene hydrate	C_10_H_18_O	Aqueous	Rhizomes	Apinundecha et al. (2023)
86	4-Terpineol	C_10_H_18_O	Aqueous	Rhizomes	Apinundecha et al. (2023)
			MeOH	Rhizomes	Tunnisa et al. (2022)
87	Geraniol	C_10_H_18_O	Aqueous	Rhizomes	Apinundecha et al. (2023)
			EtOH	Rhizomes	Kaewjai et al. (2023)
			MeOH	Roots	Chatsumpun et al. (2017)
			Hexane	Rhizomes	Liana et al. (2024)
88	Camphor	C_10_H_16_O	Aqueous	Rhizomes	Liana et al. (2024)
			Hexane	Rhizomes	Liana et al. (2024)
			EtOH	Rhizomes	Kaewjai et al. (2023)
			MeOH	Rhizomes	Nishidono et al. (2017)
89	Linalool	C_10_H_18_O	EtOH	Rhizomes	Kaewjai et al. (2023)
			MeOH	Rhizomes	Tunnisa et al. (2022)
90	Tricyclene	C_10_H_16_	Aqueous	Rhizomes	Apinundecha et al. (2023)
			MeOH	Rhizomes	Tunnisa et al. (2022)
91	α-Pinene	C_10_H_16_	Aqueous	Rhizomes	Apinundecha et al. (2023)
			MeOH	Rhizomes	Tunnisa et al. (2022)
92	β-Pinene	C_10_H_16_	Aqueous	Rhizomes	Apinundecha et al. (2023)
			MeOH	Rhizomes	Tunnisa et al. (2022)
93	α-Fenchene	C_10_H_16_	MeOH	Rhizomes	Tunnisa et al. (2022)
94	D-Camphene	C_10_H_16_	Aqueous	Rhizomes	Liana et al. (2024)
			Hexane	Rhizomes	Liana et al. (2024)
			MeOH	Rhizomes	Tunnisa et al. (2022)
			EtOH	Roots	Suwannayod et al. (2019)
95	α-Phellandrene	C_10_H_16_	Aqueous	Rhizomes	Apinundecha et al. (2023)
96	β-Phellandrene	C_10_H_16_	MeOH	Rhizomes	Tunnisa et al. (2022)
97	3-Carene	C_10_H_16_	MeOH	Rhizomes	Tunnisa et al. (2022)
			EtOH	Roots	Suwannayod et al. (2019)
			Aqueous	Rhizomes	Jantan et al. (2001)
98	cis-Limonene oxide	C_10_H_16_O	Aqueous	Rhizomes	Liana et al. (2024)
99	Limonene oxide	C_10_H_16_O	Aqueous	Rhizomes	Liana et al. (2024)
100	d-Limonene	C_10_H_16_	MeOH	Rhizomes	Tunnisa et al. (2022)
			EtOH	Roots	Suwannayod et al. (2019)
101	β-Myrcene	C_10_H_16_	Aqueous	Rhizomes	Apinundecha et al. (2023)
			MeOH	Rhizomes	Tunnisa et al. (2022)
102	trans-β-Ocimene	C_10_H_16_	Hexane	Rhizomes	Liana et al. (2024)
			Aqueous	Rhizomes	Apinundecha et al. (2023)
			MeOH	Rhizomes	Tunnisa et al. (2022)
			EtOH	Roots	Suwannayod et al. (2019)
103	α-Terpinene	C_10_H_16_	Aqueous	Rhizomes	Apinundecha et al. (2023)
			MeOH	Rhizomes	Tunnisa et al. (2022)
104	γ-Terpinene	C_10_H_16_	Aqueous	Rhizomes	Apinundecha et al. (2023)
			MeOH	Rhizomes	Tunnisa et al. (2022)
			EtOH	Roots	Suwannayod et al. (2019)
105	Neral	C_10_H_16_O	MeOH	Rhizomes	Tunnisa et al. (2022)
			Aqueous	Rhizomes	Jantan et al. (2001)
106	α-Terpineol	C_10_H_18_O	Aqueous	Rhizomes	Liana et al. (2024)
			Hexane	Rhizomes	Liana et al. (2024)
			MeOH	Rhizomes	Tunnisa et al. (2022)
107	Eucalyptol	C_10_H_18_O	Aqueous	Rhizomes	Apinundecha et al. (2023)
			MeOH	Rhizomes	Tunnisa et al. (2022)
			Hexane	Rhizomes	Liana et al. (2024)
108	Borneol	C_10_H_18_O	Aqueous	Rhizomes	Liana et al. (2024)
			MeOH	Rhizomes	Tunnisa et al. (2022)
109	o-Cymene	C_10_H_14_	MeOH	Rhizomes	Tunnisa et al. (2022)
			EtOH	Roots	Suwannayod et al. (2019)
110	(+)-4-Carene	C_10_H_16_	MeOH	Rhizomes	Tunnisa et al. (2022)
111	M-cymene	C_10_H_14_	EtOH	Roots	Suwannayod et al. (2019)
112	2-carene epoxide	C_10_H_16_O	EtOH	Roots	Suwannayod et al. (2019)
113	1,5,8-p-Menthatriene	C_10_H_14_	EtOH	Roots	Suwannayod et al. (2019)
114	Citral	C_10_H_16_O	Aqueous	Rhizomes	Liana et al. (2024)
			EtOH	Roots	Suwannayod et al. (2019)
115	Geranyl formate	C_11_H_18_O_2_	Aqueous	Rhizomes	Jantan et al. (2001)
116	Isoborneol	C_10_H_18_O	Aqueous	Rhizomes	Apinundecha et al. (2023)
117	Geranyl propionate	C_13_H_22_O_2_	Aqueous	Rhizomes	Jantan et al. (2001)
118	Neryl acetate	C_12_H_20_O_2_	Aqueous	Rhizomes	Jantan et al. (2001)
119	β-Thujaplicin	C_10_H_12_O_2_	Aqueous	Rhizomes	Jantan et al. (2001)
120	Alloocimene	C_10_H_16_	Aqueous	Rhizomes	Jantan et al. (2001)
121	α-Thujene	C_10_H_16_	Aqueous	Rhizomes	Jantan et al. (2001)
122	(Z)-β-Ocimene	C_10_H_16_	Aqueous	Rhizomes	Jantan et al. (2001)
123	Sabinene	C_10_H_16_	Aqueous	Rhizomes	Jantan et al. (2001)
124	cis-Linalool oxide	C_10_H_18_O_2_	Aqueous	Rhizomes	Jantan et al. (2001)
125	β-Citronellol	C_10_H_20_O	Aqueous	Rhizomes	Sukari et al. (2008)
126	Bicyclo (2.2.1)heptan-2-ol	C_7_H_12_O	Aqueous	Rhizomes	Sukari et al. (2008)
Alkaloids					
127	1-Octylthymine	C_13_H_22_N_2_O_2_	EtOH	Rhizomes	Chuah et al. (2022)
128	Alaptide	C_9_H_14_N_2_O_2_	EtOH	Rhizomes	Chuah et al. (2022)
129	Choline	C_5_H_13_NO	EtOH	Rhizomes	Chuah et al. (2022)
130	Hypoxanthine	C_5_H_4_N_4_O	EtOH	Rhizomes	Chuah et al. (2022)
131	Isouron	C_10_H_17_N_3_O_2_	EtOH	Rhizomes	Chuah et al. (2022)
132	Bromoxanide	C_19_H_18_BrF_3_N_2_O_4_	EtOH	Rhizomes	Chuah et al. (2022)
133	Daryamide A	C_18_H_26_N_2_O_5_	EtOH	Rhizomes	Chuah et al. (2022)
134	Levetiracetam	C_8_H_14_N_2_O_2_	EtOH	Rhizomes	Chuah et al. (2022)
135	Methylimidazoleacetic acid	C_6_H_8_N_2_O_2_	EtOH	Rhizomes	Chuah et al. (2022)
136	6-Methyladenosyl-l-methionine	C_16_H_26_N_6_O_5_S	EtOH	Rhizomes	Chuah et al. (2022)
137	DL-Arginine	C_6_H_14_N_4_O_2_	EtOH	Rhizomes	Chuah et al. (2022)
138	DL-Atenolol	C_14_H_22_N_2_O_3_	EtOH	Rhizomes	Chuah et al. (2022)
139	Emicerfont	C_22_H_24_N_6_O_2_	EtOH	Rhizomes	Chuah et al. (2022)
140	H-DL-Trp-DL-Asn-DL-Lys-DL-Lys-OH	C_27_H_42_N_8_O_6_	EtOH	Rhizomes	Chuah et al. (2022)
141	Histidyl-prolyl-tryptophan	C_22_H_26_N_6_O_4_	EtOH	Rhizomes	Chuah et al. (2022)
142	Imizol	C_22_H_26_N_6_O_3_	EtOH	Rhizomes	Chuah et al. (2022)
143	n6-Benzyladeninium nitrate	C_12_H_12_N_6_O_3_	EtOH	Rhizomes	Chuah et al. (2022)
144	Psychrophilin E	C_25_H_24_N_4_O_4_	EtOH	Rhizomes	Chuah et al. (2022)
145	N-nitroso Valsartan	C_19_H_20_N_6_O_3_	EtOH	Rhizomes	Chuah et al. (2022)
146	Oseltamivir acid	C_14_H_24_N_2_O_4_	EtOH	Rhizomes	Chuah et al. (2022)
147	Pilocarpine	C_11_H_16_N_2_O_2_	EtOH	Rhizomes	Chuah et al. (2022)
148	N-Methyl-L-histidine	C_7_H_11_N_3_O_2_	EtOH	Rhizomes	Chuah et al. (2022)
149	Triisopropanolamine	C_9_H_21_NO_3_	EtOH	Rhizomes	Chuah et al. (2022)
150	Tetracyanoindane	C_13_H_6_N4	EtOH	Rhizomes	Chuah et al. (2022)
151	Terrazoanthine B	C_21_H_24_N_6_O_2_	EtOH	Rhizomes	Chuah et al. (2022)
152	Topixantrone	C_21_H_26_N_6_O_2_	EtOH	Rhizomes	Chuah et al. (2022)
153	Z-Lys (boc)-ome	C_20_H_30_N_2_O_6_	EtOH	Rhizomes	Chuah et al. (2022)
154	Tosedostat	C_21_H_30_N_2_O_6_	EtOH	Rhizomes	Chuah et al. (2022)
155	Tetraxetan	C_16_H_28_N_4_O_8_	EtOH	Rhizomes	Chuah et al. (2022)
156	Val-Trp-His	C_22_H_28_N_6_O_4_	EtOH	Rhizomes	Chuah et al. (2022)
Other aromatic metabolites					
157	Demethoxyyangonin	C_14_H_12_O_3_	DCM	Rhizomes	Kabir et al. (2020)
			MeOH	Roots	Rungsa et al. (2023)
158	Dihydro-5,6-dehydrokawain	C_14_H_14_O_3_	CHCl_3_	Rhizomes	Tuchindaa et al. (2002)
159	Benzaldehyde	C_11_H_12_O_3_	EtOH	Rhizomes	Chuah et al. (2022)
160	Benzoylacetone	C_7_H_6_O	EtOH	Rhizomes	Chuah et al. (2022)
161	Myristicin	C_10_H_10_O_2_	Hexane	Rhizomes	Jantan et al. (2001)
162	3′,4′-Dimethoxyacetophenone	C_10_H_12_O_2_	Aqueous	Rhizomes	Sukari et al. (2008)
163	Rosephenone	C_10_H_9_Cl_3_O_2_	Aqueous	Rhizomes	Sukari et al. (2008)
164	Ethyl benzoate	C_9_H_10_O_2_	Aqueous	Rhizomes	Sukari et al. (2008)
165	Geranyl benzoate	C_17_H_22_O_2_	Aqueous	Rhizomes	Sukari et al. (2008)
166	Cinnamyl cinnamate	C_18_H_16_O_2_	Aqueous	Rhizomes	Sukari et al. (2008)
167	Panduratin H	C_20_H_26_O_2_	EtOH	Rhizomes	Win et al. (2008)
168	Panduratin I	C_20_H_26_O_2_	EtOH	Rhizomes	Win et al. (2008)
169	γ-phenylbutyric acid	C_10_H_12_O_2_	EtOH	Roots	Suwannayod et al. (2019)
Phenols					
170	Esculetin	C_9_H_6_O_4_	EtOH	Rhizomes	Chuah et al. (2022)
171	Phloroglucinol	C_6_H_6_O_3_	EtOH	Rhizomes	Chuah et al. (2022)
172	Resveratrol	C_14_H_12_O_3_	EtOH	Rhizomes	Chuah et al. (2022)
173	Vestitol	C_16_H_16_O_4_	EtOH	Rhizomes	Chuah et al. (2022)
174	Guaiacol	C_7_H_8_O_3_	Aqueous	Rhizomes	Sukari et al. (2008)
175	Caffeic acid	C_9_H_8_O_4_	MeOH	Rhizomes	Ling et al. (2010)
176	ρ-Coumaric acid	C_9_H_8_O_3_	MeOH	Rhizomes	Ling et al. (2010)
177	Chlorogenic acid	C_16_H1_8_O_9_	MeOH	Rhizomes	Ling et al. (2010)
Amides					
178	Capsi-amide	C_17_H_35_NO	EtOH	Rhizomes	Chuah et al. (2022)
179	Amide C18	C_18_H_37_NO	EtOH	Rhizomes	Chuah et al. (2022)
180	Hexadecanamide	C_16_H_33_NO	EtOH	Rhizomes	Chuah et al. (2022)
181	Lauramide	C_12_H_25_NO	EtOH	Rhizomes	Chuah et al. (2022)
182	Oleamide	C_18_H_35_NO	EtOH	Rhizomes	Chuah et al. (2022)
183	Pipericine	C_22_H_41_NO	EtOH	Rhizomes	Chuah et al. (2022)
Lipids					
184	trans-2-Hexenyl propionate	C_9_H_16_O_2_	Aqueous	Rhizomes	Sukari et al. (2008)
185	Cyclohexyl propionate	C_9_H_16_O_2_	Aqueous	Rhizomes	Sukari et al. (2008)
186	Methyl-n-nonanoate	C_10_H_19_O_2_	Aqueous	Rhizomes	Sukari et al. (2008)
187	2-Cyclohexylethyl acetate	C_10_H_18_O_2_	Aqueous	Rhizomes	Sukari et al. (2008)
188	N-Hexyl angelate	C_11_H_20_O_2_	Aqueous	Rhizomes	Sukari et al. (2008)
Organic acids					
189	Dichloroacetic acid	C_2_H_2_C_l2_O_2_	EtOH	Rhizomes	Chuah et al. (2022)
190	Sorbic acid	C_6_H_8_O_2_	EtOH	Rhizomes	Chuah et al. (2022)
191	Valerenic acid	C_15_H_22_O_2_	EtOH	Rhizomes	Chuah et al. (2022)
Diterpenes					
192	Seikphoochinal A	C_18_H_24_O	CHCl_3_	Rhizomes	Win et al. (2019)
193	Galanals A	C_20_H_30_O_3_	CHCl_3_	Rhizomes	Win et al. (2019)
194	Galanals B	C_20_H_30_O_3_	CHCl_3_	Rhizomes	Win et al. (2019)
Other metabolites					
195	N-Hexanal	C_6_H_12_O	Aqueous	Rhizomes	Sukari et al. (2008)
196	1,3-Tetradecadiene	C_14_H_26_	Aqueous	Rhizomes	Sukari et al. (2008)
197	2-Hexenal	C_6_H_10_O	Aqueous	Rhizomes	Liana et al. (2024)
198	n-Butyl-n-pentadecanoate	C_19_H_38_O_2_	Aqueous	Rhizomes	Sukari et al. (2008)
199	n-Eicosane	C_20_H_42_	Aqueous	Rhizomes	Sukari et al. (2008)
200	(Z) 6-Pentadecen-1-ol	C_15_H_30_O	Hexane	Rhizomes	Liana et al. (2024)
201	Simvastatin	C_25_H_38_O_5_	EtOH	Rhizomes	Chuah et al. (2022)
202	Dichlormid	C_8_H_11_Cl_2_NO	EtOH	Rhizomes	Chuah et al. (2022)
203	Acetyl chloride	C_2_H_3_ClO	EtOH	Rhizomes	Chuah et al. (2022)
204	Phosphanyl (phospholan-1-yl) phosphane	C_4_H_11_P_3_	EtOH	Rhizomes	Chuah et al. (2022)
205	2-Isopropyl-4,5-dimethyloxazole	C_8_H_13_NO	Aqueous	Rhizomes	Sukari et al. (2008)

CO2 SFE, carbon dioxide supercritical fluid extraction; EtOH, ethyl alcohol; DCM, dichloromethane; MeOH, methanol; CHCl_3_, trichloromethane.

### 5.1 Flavonoids

Flavonoid metabolites are widely present in plants, and their special structures confer significant activity (Dhiman et al., 2016; Murti et al., 2021). Within the context of *B. rotunda*, flavonoids and their derivatives are recognized as the predominant pharmacologically active metabolites, exhibiting a prolific presence. To date, a cumulative total of 83 flavonoids and their derivatives (**1–83**) have been successfully isolated from *B. rotunda*. In recent years, these flavonoids have attracted considerable research interest, with studies revealing an impressive spectrum of biological activities. In a recent study, Thadtapong et al. (2024) identified that metabolite 20 potently inhibits multidrug-resistant *Acinetobacter baumannii*, showcasing remarkable anti-bacterial properties. Furthermore, Liana et al. (2024) reported that the flavonoid metabolite Pinostrobin (**1**), isolated from *B. rotunda*, exerts a significant inhibitory effect on atopic dermatitis. This finding implies the potential of Pinostrobin (**1**) as a topical skincare product or therapeutic agent for the management of this skin condition. These findings underscore the therapeutic potential of *B. rotunda*’s flavonoids and highlight the importance of continued research into their medicinal applications.

### 5.2 Monoterpenes

Monoterpene metabolites are ubiquitous in the plant kingdom and are important metabolites of plant essential oils. Although the structure is simple, it has significant biological activity (Liu et al., 2021). To date, approximately 43 distinct monoterpenes (**84–126)**, have been identified in *B. rotunda*, serving as the principal determinants of its characteristic fragrance. Presently, research on monoterpene metabolites is predominantly concentrated on essential oil mixtures, which has resulted in a significant knowledge gap regarding the study of individual monoterpene metabolites.

### 5.3 Alkaloids

In recent years, it has been found that alkaloid metabolites have good biological activity and can effectively treat various diseases (Kittakoop et al., 2022). To date, approximately 30 alkaloids (**127–156**), have been isolated and purified from *B. rotunda*, predominantly from its rhizomes. Nonetheless, the investigation of alkaloids in *B. rotunda* is still in its infancy, with a notable dearth of comprehensive studies exploring their pharmacological potential.

### 5.4 Other aromatic metabolites

Additionally, *B. rotunda* is noted for its aromatic metabolites that incorporate a benzene ring structure. Thus far, approximately 13 distinct aromatic metabolites (**157–169**), have been identified within the rhizomes of *B. rotunda*. However, research pertaining to these specific metabolites is sparse, indicating a need for further investigation. Broadening the research horizon of *B. rotunda* necessitates an intensified investigation into the active aromatic metabolites present within this species.

### 5.5 Phenols

Phenolic metabolites are widely used in the field of pharmaceutical cosmetics because of their wide range of pharmacological activities (Alu’datt et al., 2016). To date, 8 phenolic metabolites (**170–177**), have been successfully isolated from *B. rotunda*. However, the biological effects of these phenolic metabolites remain inadequately explored, and the specific biological activities of these metabolites are yet to be fully elucidated. This underscores an urgent requirement for additional research endeavors to uncover their potential therapeutic applications.

### 5.6 Amides

In recent years, amide metabolites have become an indispensable part of pharmacology due to their diverse biological activities (Narendar Reddy et al., 2019). In the context of *B. rotunda*, approximately six fatty alcohol metabolites (**178–183**), have been identified across various parts of the plant. Despite this, the current body of research regarding the biological activities of these specific metabolites is sparse, indicating a significant gap that warrants further exploration and investigation. This underscores the need for a more in-depth analysis of these metabolites to elucidate their potential roles and applications in pharmacology and medicine.

### 5.7 Lipids

Naturally occurring lipid metabolites, which incorporate ester functionalities into their molecular structures, play a pivotal role in a multitude of biological processes. These metabolites are characterized by their unique physical and chemical properties, which are essential for various life functions. Within *B. rotunda*, five ester metabolites (**184–188**) have been identified, contributing to the plant’s metabolic profile. Despite their identification, the biological activities of these ester metabolites remain largely unexplored, indicating a significant gap in our understanding of their potential roles and applications.

### 5.8 Organic acids

Organic acid metabolites are widely present in plants. They play an important role in various industrial and biological applications (Yan et al., 2024). At present, three types of organic acid metabolites (**189–191**) have been found from *B. rotunda*. Despite their identification, there is a dearth of extensive research on the biological activities of these organic acid metabolites, suggesting a significant area for future investigative efforts.

### 5.9 Diterpenes

Currently, *B. rotunda* has been identified to contain three diterpenoids, namely, metabolites **192–194**. Notably, metabolites **193** and **194** have demonstrated significant anti-proliferative effects on human cancer cell lines, as reported by Win et al. (2019). The identification of these bioactive diterpenoids in *B. rotunda* suggests that they may play a crucial role in the plant’s chemotherapeutic applications, warranting in-depth investigation into their potential as novel cancer therapeutics.

### 5.10 Other metabolites

In addition to the well-documented diterpenoids and other metabolites, *B. rotunda* has yielded a diverse array of organic metabolites, including those designated as **195–205**, which have been identified across various parts of the plant. Despite the potential health-promoting effects suggested by their presence, the scientific literature on the specific health benefits of these metabolites is currently limited. This underscores an urgent research imperative to explore the bioactivity and potential applications of these organic metabolites within the context of *B. rotunda*.

## 6 Pharmacological activities

**FIGURE 4:**
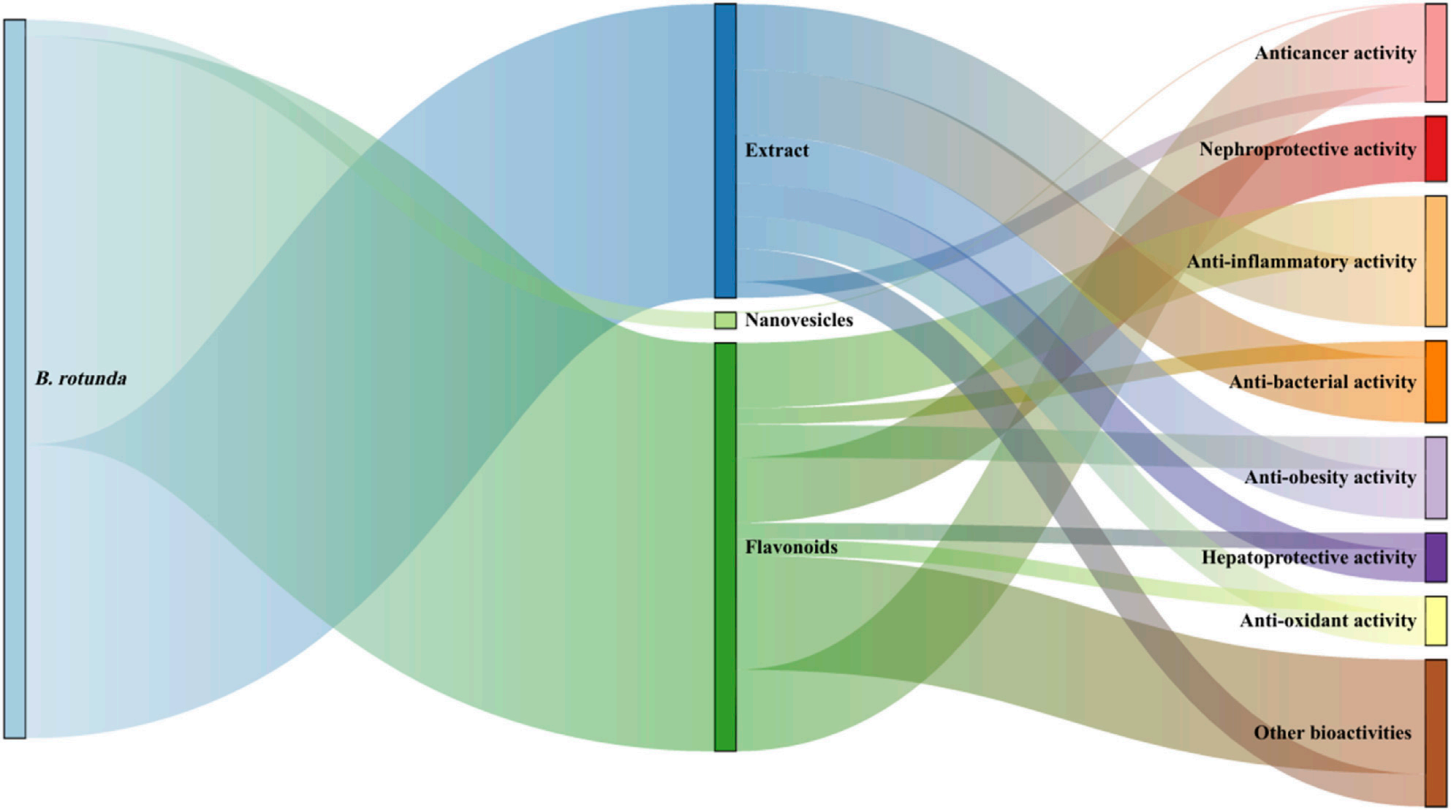
Sankey diagram of pharmacological activities of *B. rotunda*.

**FIGURE 5:**
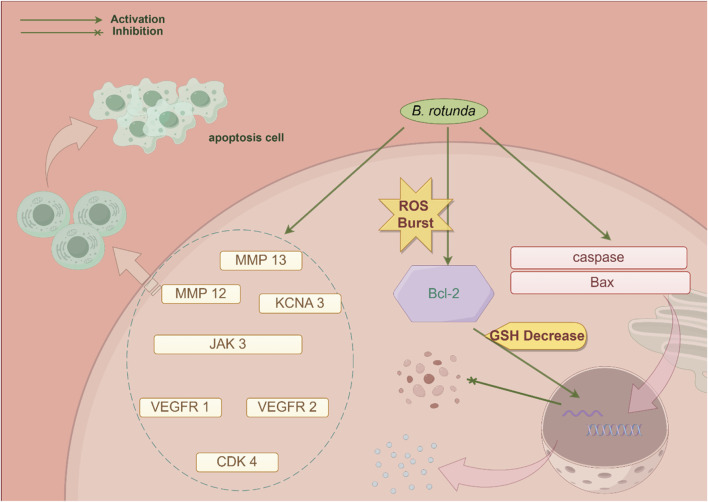
A conceptual diagram illustrating the potential mechanism underlying the anti-cancer activity of *B. rotunda* is presented.

**FIGURE 6:**
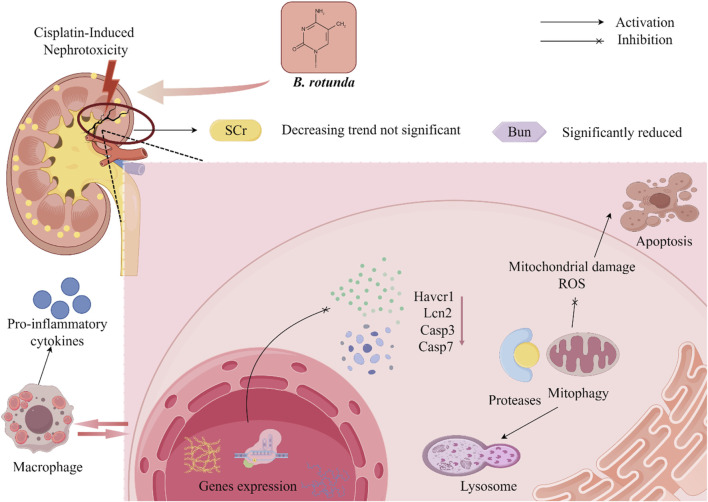
A diagrammatic depiction of the plausible mechanism underlying the nephroprotective activity of *B. rotunda* is provided.

**FIGURE 7:**
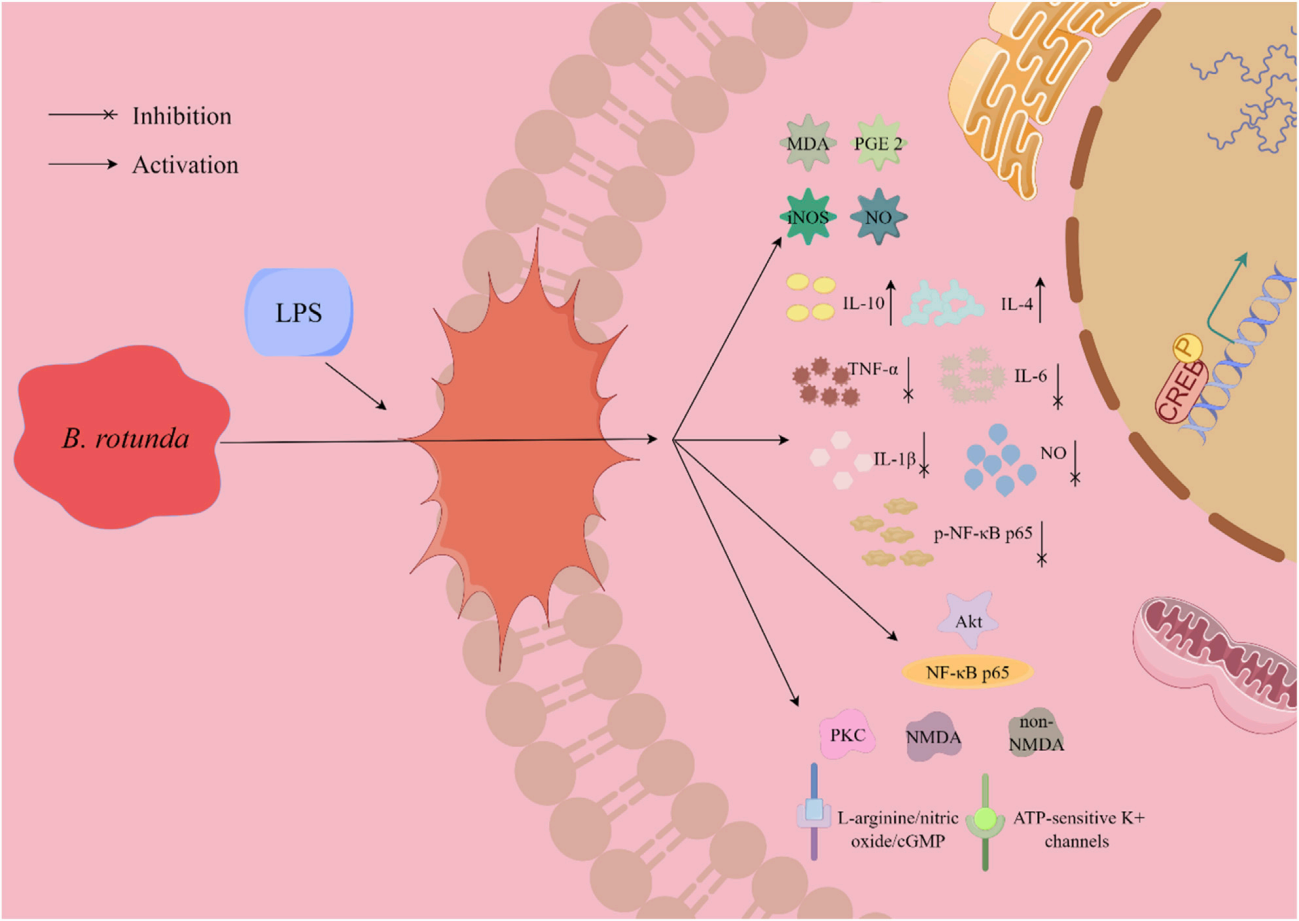
A graphical illustration depicting the potential mechanism underlying the anti-inflammatory effects of *B. rotunda* is presented.

At present, it has been found to have significant biological activity from the extracts and metabolites of *B. rotunda*. This suggests that *B. rotunda* can be widely used to prevent and improve a range of diseases. It has been found that *B. rotunda* has many other potential therapeutic effects such as anti-tumor, anti-cancer, anti-inflammatory, anti-bacterial, anti-oxidation and anti-obesity. The manifold biological activities of *B. rotunda* will be vividly delineated through a Sankey diagram, providing a visual synthesis of its multifaceted therapeutic potential (Figure 4) (Feng et al., 2024). Furthermore, Figures 5–7 elucidate the potential mechanisms that underpin the partial biological activities of *B. rotunda* extracts and their bioactive metabolites. A comprehensive overview of these metabolites and their associated bioactivities is presented in Table 2, with numerical references aligning with those in Table 1, which enumerates the names of each metabolite. These pharmacological findings of *B. rotunda* were further followed up with an exhaustive review and in-depth discussion to delve into its multiple biological activities.

**TABLE 2:** Summary of biological activities of bioactive metabolites and extracts of *Boesenbergia rotunda* (L.). (Increase, ↑; Decrease, ↓).

Biological activities	Name	Types	Testing subjects	Doses/Duration	Effects/Mechanisms	References
Anti-cancer activity						
	BRE	*In vitro*	Breast cancer cell line T47D and human fibroblast cell line TIG-1	0, 10, 20, 40, and 80 μg/mL for 24 h	Number of T47D cells undergoing apoptosis and cells lost MMP ↑;	Widyananda et al. (2022)
	FDNVs	*In vitro*	Colorectal cancer HT-29 and HCT116 cell lines	3.13, 6.25, 12.5, 25,50, and 100 μg/mL for 72 h	Cell apoptosis ↑; caspase-3, caspase-9, Bax and expression ↑; Bcl-2 expression ↓; ROS concentrations ↑; GSH level ↓;	Chuah et al. (2022)
	27	*In vitro*	Nasopharyngeal Carcinoma Cells (HK1 and NP69 cell)	0, 3.125, 6.25, 12.5, 25, 50, 100 and 200 μg/mL for 48 h	Cell viability and migration ↓; apoptosis ↑;	Break et al. (2020)
	20	*In vitro*	Human malignant melanoma A375 cells	7.5, 15, and 30 μM for 24 h	Caspase-3 activity and cleaved PARP ↑; cell death ↑; mitochondria ↑; mitochondrial potential ↓; ATP production ↓; phosphorylation of eIF2α and expression of CHOP ↑; Bcl-2 protein and cytochrome ↑;	Lai et al. (2015)
	20	*In vitro*	A549 human non-small cell lung cancer cells.	1.25, 2.5, and 5 μg/mL for 24 h	Caspase-3 activity ↑; NF-κB/p65 and NF-κB/p50 nuclear levels ↓; p53 and p21 ↑;	Cheah et al. (2013)
	35	*In vitro*	Human T4-lymphoblastoid cell line	3.125, 5, 6.25, 8, 10, 12.5,25, 50, and 100 μg/mL for 72 h	Caspases −3/7, −8 and −9 activities ↑;	Ng et al. (2013)
	20	*In vitro*	A549 human non-small cell lung cancer cells	0.625, 1.25, 2.5, and 5 μg/mL for 72 h	Cell metabolic activity ↓; cell number ↓; plasma membrane permeability, fluorescence intensity of nuclei, factin content and mitochondrial mass/pontential ↑; NF-κB translocation ↓;	Cheah et al. (2011)
Nephroprotective activity						
	35	*In vivo*	Cisplatin-induced acute kidney injury SD rats	125, 250, and 500 mg/kg BW, i.g., once daily, for 10 days	Creatinine, urea nitrogen, glutamic pyruvate transaminase, and malondialdehyde levels ↓; expression of KIM-1, NGAL, Casp3, and Casp7 genes ↓;	Sujana et al. (2024)
	20	*In vivo*	Male C57BL/6 mice	2.5 and 25 mg/kg BW, i.p., for 7 days	Renal tubular degeneration, blood BUN levels, expression of renal injury biomarker, and apoptosis proteins ↑; oxidative stress and apoptosis in kidney tissues ↓;	Worakajit et al. (2022)
	20	*In vitro*	Human renal proximal tubular cells (RPTEC/TERT1)	2.5 and 5 µM for 72 h	Cell apoptosis, elevated ROS production ↓; mitochondrial membrane potential ↑; Ngal, Tim-1, cytochrome, and cleaved-caspase 3 expressions ↓; Bcl-2 expression ↑;	Worakajit et al. (2022)
	1	*In vivo*	Mature male SD rats	5 and 10 mg/kg BW, i.g., once daily, for 30 days	SOD, GSR, CAT, and GSH-PX enzymes ↑; MDA and ROS levels ↓; urea, KIM-1, NGAL, and creatinine levels ↓; IL-1β, NF-κB, IL-6, TNF-α levels and iNOS and COX-2 activity ↓; Bcl-2, caspase-3, and Bax expression ↑; MDH, ICdH, α-KGDH, and SDH enzymes activities ↑;	Ijaz et al. (2023)
Anti-inflammatory activity						
	BRE	*In vitro*	RAW 264.7 macrophages	15, 30, and 60 μg/mL for 1 h	IL-1β, TNF-α, and NO levels ↓;	Taechowisa et al. (2024)
	20	*In vitro*	LPS-stimulated SIMA9 microglial cells	0.1, 1, 5, 10, and 50 μM for 48 h	NO production, iNOS expression, and p-NF-κB p65 levels ↓; TNF-α, IL-1β, and IL-6 mRNA expression ↓; IL-4 and IL-10 mRNA expression levels ↑;	Jamornwan et al. (2022)
	20	*In vivo*	18-month-old male beagle dogs	1 mg/kg BW, i.v., once daily; 5 and 10 mg/kg BW, p.o., once daily, for 7 days	C_max_ and AUC ↑;	Boonyarattanasoonthorn et al. (2023)
	20	*In vivo*	SARS-CoV-2-infection in Eight-week-old male Crl:LVG (SYR) hamsters	300 and 1,000 mg/kg BW 7 days	Body weight ↑; PGE2 and IL-6concentrations ↓;	Kongratanapasert et al. (2023)
	EBR	*In vivo*	Combined induction of diabetic peripheral neuropathy by 30% fructose solution and streptozotocin in SD rats	100 and 400 mg/kg BW for 5 weeks	Blood glucose concentration and body weight ↓; thermal hyperalgesia, and cold ↓; mechanical allodynic responses ↑; TNF-α and IL-1β ↓;	Wang et al. (2022)
	BREE	*In vivo*	Wistar albino rats	20, 250, 500.1,000, 2000, and 4,000 mg/kg BW, i.g., for 18 h	Infiltration ↓; Akt expression and NF-κB p65 ↓;	Rosdianto et al. (2020)
	BRE	*In vitro*	DPPH free radicals and NO assay	31.25–1,000 μg/mL for 30 min	DPPH and NO scavenging capacity ↑;	Widyananda et al. (2022)
	35	*In vivo*	Ethanol-induced ulcer model in rats	10 and 20 mg/kg BW, i.g., for 1 h	GSH, MDA, and NO level ↓; NP-SH level ↑; TNF-α, IL-6, and iNOS ↓; PGE2 and HSP ↑;	Mohan et al. (2020)
Anti-bacterial activity						
	20	*In vitro*	*A. baumannii* Aci44 and Aci46	1, 2.5, and 5 μM for 24 h	MIC and MBC of colistin ↓; metabolic activities in biofilm and biofilm mass ↓; oxidoreductase, superoxide dismutase, thiol peroxidase, NADH ubiquinone oxidoreductase, ATP synthase gene expression ↓; IscU, iron transporter, and ferrichrome-iron receptor gene ↓; *bla*_*OXA-23*_ expression ↑; *tetA* expression ↓; ROS production ↑;	Thadtapong et al. (2024)
	BREO	*In vitro*	MRSA isolates and *Staphylococcus aureus* ATCC 29213	1, 2, and 4 mg/mL for 18 h	OD_260_ of cells ↑; cytoplasmic membrane permeability and bioflm inhibition activity↑;	Apinundecha et al. (2023)
	BRE	*In vitro*	*C. albicans* ATCC 10231	3.125, 6.25, 12.5, 50, 100, and 200 μg/mL for 72 h	Biofilm formation and cell surface hydrophobicity ↓; *ACT1* mRNA level and *ALS3* mRNA expression↓;	Kanchanapiboon et al. (2020)
	RH3.5	*In vitro*	36 actinomycete strains	15, 30, and 60 μg/mL for 15 days	Inhibition of actinomycete ↑; the growth of *S. aureus* ↓;	Taechowisa et al. (2024)
	BRE	*In vitro*	*Staphylococcus aureus* and *Staphylococcus epidermidis*	0.25, 0.5, 1, 2, 6, 8, and 16 μg/mL for 24 h	CLX, AMP and CFZ ↓; average cell areas ↓;	Teethaisong et al. (2018)
	BRE	*In vitro*	A clinical isolate *Blastocystis hominis* subtype 3	62.5, 125, 250, and 500 μg/mL for 24 h	Growth capacity and number ↓; cell shrinkage ↑;	Kaewjai et al. (2023)
Anti-obesity activity						
	21	*In vitro*	Human PCS-210–010 preadipocyte and mouse embryonic preadipocyte 3T3-L1 cells	1, 5, and 10 μM for 72 h	Intracellular triglyceride levels ↓; extracellular glycerol levels ↑; FAS, PLIN, LPL, adiponectin, SREBP-1c, PPARγ, and C/EBPα expression ↓; MCE-mediated protein expression ↓; SREBP-1C, PPARγ, and C/EBPα expression levels ↓; p-JNK/JNK, p-ERK/ERK, and p-p38/p38 levels ↓; p-ACC/ACC, p-AMPKα/AMPKα, and p-AMPKβ/AMPKβ levels ↑; AKT/GSK3β and AKT/AMPK-ACC expression ↓; adipogenesis ↓;	Rungsa et al. (2023)
	1	*In vitro*	Human PCS-210–010 preadipocyte and mouse embryonic preadipocyte 3T3-L1 cells	1, 5, 10, 20, 50, and 100 μM for 8 days	C/EBPα, PPARγ, and SREBP-1c mRNA and protein levels ↓; p-Akt and p-GSK3β expression ↓; p-ACC/ACC level ↑; p-JNK and p-p38 expression ↓; p-JNK/JNK and p-p38/p38 levels ↓; cellular lipid content ↓;	San et al. (2022)
	BP-3	*In vitro*	3T3-L1 preadipocyte cells	1,5,10,20, and 50 μg/mL for 8 days	Lipid droplets size and number ↓; triglyceride accumulation ↓;	Jitsaeng et al. (2023)
	BRE	*In vivo*	High fructose/streptozotocin-induced diabetic male SD rats	100 and 400 mg/kg BW, i.g., for 5 weeks	Fasting blood glucose level, HbA1c, lipid profile, hepatorenal biochemical parameters, and IPGTT ↓; body weight ↑; food and water intake, creatinine, BUN and uric acid levels, AST, ALT and ALP levels, serum total cholesterol, triglycerides and LDL-c, pancreatic β-cell function, and pancreatic cell morphology ↓; HDL ↑; fructose 1,6 biphosphatase, glucose-6-phosphatase, malondialdehyde, and proinflammatory cytokines levels ↓; GSH, SOD, and CAT enzymes activities ↑; TNF-α and IL-1β levels ↓;	Olatunji et al. (2022)
	BRE	*In vitro*	DPPH and ABTS	10 mg/mL for 24 h	DPPH and ABTS scavenging activity ↑;	Olatunji et al. (2022)
Hepatoprotective activity						
	20	*In vivo*	SD rat model of cirrhosis induced by thioacetamide	5,10, and 50 mg/kg BW, i.g., for 8 weeks	Serum PDGF and TGF-b1 ↓; hepatic MMP-2 and TIMP-1 ↓; oxidative stres ↓; AP, ALT, AST, and GGT levels ↓; OH-dG, NT, and MDA levels ↓; SOD, CAT, and GPx levels ↑; PDGF and TGF-β1 ↓;	Salama et al. (2018)
	BRE	*In vivo*	Thioacetamide-induced liver damage in *Sprague-Dawley* rats	50, 250, and 500 mg/kg BW, i.g., for 8 weeks	Hepatic Level of CYP2E1 ↓; liver nitrotyrosine and urinary 8-OH-dG levels ↓; CAT and GPx levels ↑; TGF-*β*1, NF-*κ*B, and IL-6 ↓; Bax and caspase-3 ↑; Bcl-2, MMP-2, MMP-9, and TIMP-1 level ↓; SOD, Gpx, and CAT ↑; OH-dG, MDA, and NT ↓;	Salama et al. (2013)
	BRE	*In vivo*	Thioacetamide-Induced Liver Cirrhosis in *Sprague-Dawley* rats	2 and 5 g/kg BW, i.g., for 8 weeks	MDA levels ↓; SOD ↑; liver enzymes AP, ALT, AST, LDH, cholesterol, LDL, and triglycerides levels ↓	Salama et al. (2012)
Anti-oxidant activity						
	MEBR	*In vivo*	Adult male and female SD rats	50, 100, 200, and 400 mg/kg BW, for 14 days	Ulcer area, mucosal content, submucosal edema, and leucocytes infiltration ↓;	Abdelwahab et al. (2011)
	MEBR	*In vitro*	RAW 264.7 cells	0.81, 1.78, 3.13, 6.25, 12.5, 25, and 50 μM for 24 h	TBARS level ↑; NO level ↓;	Abdelwahab et al. (2011)
	20	*In vitro*	DPPH scavenging and human embryonic normal liver cell line (WRL-68)	1, 10 and 100 μg/mL for 72 h	DPPH scavenging activity and the ferric reducing anti-oxidant power ↑; Protective effects of cytotoxicity ↑; protein MDA level ↓; CAT, SOD and GPx levels ↑;	Salama et al. (2013)
Other bioactivities						
	1	*In vivo*	Induce PD in male Wistar rat	40 mg/kg BW, i.g., once daily, for 7 days	Motor coordination ↑; MDA content ↓; GSH SOD, and DA levels ↑; the death of principal neurons and TH-positive neurons ↓; the expression of GDNF ↑;	Kongsui et al. (2023)
	1	*In vivo*	Adult male Wistar rats	20 and 40 mg/kg BW, i.g., once daily, for 21 days	MDA and anti-oxidant enzymes levels ↓; COD and CAT activities ↑; recognition index ↑; the density of surviving cells in the CA1 and CA3 regions ↑; GFAP ↑; EAAT2 expression ↑;	Thongrong et al. (2023)
	1	*In vivo*	Male Wistar rats of sciatic nerve crush injury	20 and 40 mg/kg BW for 28 days	GSH sulfhydryl molecules ↑; MDA level ↓; DRG neurons ↑; axon diameter ↑; p-ERK1/2 level ↑;	Kongsui et al. (2023)
	20	*In vivo*	BALB/c mice	5 μM, i.p., for 7 days	Hemoglobin content and neo-vascularization ↓;	Bereswill et al. (2012)
	20	*In vivo*	Zebrafish	15 μM for 24 h	Neo-vascularization ↓;	Bereswill et al. (2012)
	20	*In vitro*	Human umbilical vein endothelial cells	3.5, 7, and 14 mM for 16h	Cytostatic and cytotoxic effects↑; chemotactic invasiveness and percentage of migrated cells ↓;	Bereswill et al. (2012)
	2	*In vitro*	Human keratinocyte cell line	15.6, 31.3, 62.5, 125, and 500 µM for 0 min, 2 min, 5 min, 0.25 h, 0.5 h, 1 h, 3 h, 6 h, 12 h, and 24 h	Cell viability, cellular wound healing rate, and cell number of keratinocytes ↑; ERK1/2 and Akt activity ↑;	Ruttanapattanakul et al. (2022)
	BRE (kaempferol)	*In vitro*	Human keratinocyte cell line	0.2, 0.5, 0.9, 1.9, 3.9, 7.8, 15.6, 31.3, 62.5, 125, and 250 μg/mL for 48 h	Cell number and migration ↑; ERK1/2 and Akt phosphorylation ↑;	Ruttanapattanakul et al. (2021)
	BRE	*In vivo*	Seven weeks old adult male SD rats	100 and 400 mg/kg BW, i.p., once daily, for 7, 14, and 21 days	AST, ALP, LDH, TnT, and CK-MB levels ↓; the characteristic indices ↓; p53 and MPO proteins ↓;	Zhang et al. (2023)

**Notes:** BRE, *Boesenbergia rotunda* extract; MEBR, methanolic extract of *B. rotunda*; BP-3, *B. rotunda* in fraction 3; BREE, the ethanol extract of *B. rotunda* rhizome; EBR, *B. rotunda* extract; FDNVs, fingerrootderived (*B. rotunda*) nanovesicles; BREO, *B. rotunda* essential oil.

### 6.1 Anti-cancer activity

In their comprehensive analysis of *B. rotunda* utilizing the KNApSAcK database, Widyananda et al. (2022) identified the presence of 20 metabolites, with chalcones and flavonoids being the most prevalent. Among these identified metabolites, seven metabolites have exhibited anti-cancer properties. These include Sakuranetin (**19)**, Cardamonin (**27)**, Alpinetin (**3)**, Pinocembrin (**2)**, 7,4′-Dihydroxy-5-methoxyflavanone (**8)**, Demethoxyyangonin (**157)**, and Pinostrobin chalcone (**28)**. Utilizing predictive analysis, these metabolites have demonstrated stable interactions with key proteins, including matrix metallopeptidase 12 (MMP 12), cyclin-dependent kinase 4 (CDK 4), janus kinase 3 (JAK 3), vascular endothelial growth factor receptor 2 (VEGFR 2), matrix metallopeptidase 13 (MMP 13), vascular endothelial growth factor receptor 1 (VEGFR 1), and potassium voltage-gated channel subfamily A member 3 (KCNA 3). These interactions suggest a potential for these metabolites to inhibit proliferation and induce apoptosis in breast cancer cells. Additionally, *in vitro* assays have confirmed that *B. rotunda* extract exerts cytotoxic effects on T47D cells, initiating apoptotic pathways. On a global scale, colorectal cancer (CRC) remains one of the most prevalent forms of cancer, posing significant threats to human health and survival. Chuah et al. (2022) conducted a study examining the therapeutic efficacy of nanovesicles derived from fingerroot (FDNVs) in the context of CRC, uncovering significant anti-cancer activity against the CRC cell lines HT-29 and HCT 116. The treatment with 25 μg/mL of *B. rotunda* extract for a duration of 24 h led to pronounced cytotoxicity in all cell lines under investigation. Consistently, FDNVs elicited cytotoxic effects on both CRC cell lines in a dose- and time-dependent manner, with inhibitory concentration 50 (IC_50_) values of 47.8 and 34 μg/mL achieved after a 72 h exposure, respectively. Notably, FDNVs displayed no cytotoxicity towards normal human colonic epithelial cells. Moreover, FDNVs triggered early apoptosis in HT-29 and HCT116 cells in a dose-responsive fashion, upregulating the expression of cysteinyl asparaginase and the pro-apoptotic gene bcl-associated X protein (Bax), while downregulating the expression of the anti-apoptotic B-cell lymphoma-2 (Bcl-2) gene. These findings suggest a selective anti-cancer effect of FDNVs on CRC cells, highlighting their potential as a targeted therapeutic agent.

Additionally, Break et al. (2020) reported that Cardamonin (**27**), a chalcone metabolite derived from *B. rotunda*, exerted notable cytotoxic effects on Nasopharyngeal Carcinoma Cells (HK1), with an IC_50_ value of 26.7 mg/mL achieved after a 48 h treatment period. In stark contrast, the IC_50_ value against normal NP69 cells exceeded 200 mg/mL, highlighting a significantly higher selectivity for HK1 cells. This finding suggests a marked preference for cancer cells with minimal effects on normal cellular counterparts. Western blot analysis revealed that myricetin did not significantly modulate the expression levels of B-cell lymphoma-extra large (Bcl-XL), Bcl-2, or Bax, leading to the hypothesis that Cardamonin (**27**) induces apoptosis in HK1 cells via an extrinsic, rather than intrinsic, apoptotic pathway. Cheah et al. (2011) reported that Panduratin A (**20**), a bioactive metabolite isolated from *B. rotunda*, significantly inhibited the proliferation of A549 human non-small cell lung cancer cells. This metabolite exhibited cytotoxicity against these cells, with a half maximal IC_50_ of 4.4 μg/mL, which is equivalent to 10.8 μM. Further studies demonstrated Panduratin A’s (**20**) capacity to induce G2/M phase arrest in cancer cells, resulting in the accumulation of BrdU-labeled cancer cells and an increase in phosphorylated histone H3 levels. Simultaneously, Panduratin A (**20**) led to a decrease in cancer cell viability and an enhancement in plasma membrane permeability. These observations suggest a significant inhibitory effect on the activation of the nuclear factor-κB (NF-κB) signaling pathway, which may underlie its anti-cancer activity.

Melanoma, a highly aggressive cutaneous tumor originating from melanocytes, is characterized by its propensity for metastasis and its significant impact on patient prognosis. Lai et al. (2015) investigated the biological effects of Panduratin A (**20**), a cyclohexyl chalcone metabolite isolated from *B. rotunda*, on human malignant melanoma A375 cells. Preliminary findings revealed that Panduratin A (**20**) possessed cytotoxic activity against A375 cells, with an IC_50_ value of 11.66 μM. Furthermore, the primary target of Panduratin A (**20**) in A375 cancer cells was identified as the mitochondria, suggesting its role in modulating mitochondrial oxidative phosphorylation, secretion, and endoplasmic reticulum stress pathways. The metabolite was found to induce apoptosis through sustained endoplasmic reticulum stress via the PERK/eIF2α/ATF4/CHOP signaling cascade.

The flavonoid and polyphenol metabolites present in extracts of *B. rotunda* have demonstrated potent inhibitory effects on tumor and cancer cells. Experimental data have underscored the therapeutic potential of *B. rotunda* in addressing breast, skin, and digestive system malignancies. Nonetheless, the correlation between the chemical structures of these metabolites and their biological activities is an area that is not well-explored, and the precise mechanisms underlying their anti-cancer effects remain to be fully elucidated. Furthermore, the innovative role of these chemical metabolites in the specific targeting of tumors or cancer cells warrants additional investigation. Future research endeavors should concentrate on deciphering the structure-activity relationship of these metabolites, thoroughly examining the bioactivity of flavonoid and polyphenol metabolites, and dissecting their mechanisms of action to augment their therapeutic efficacy and specificity.

### 6.2 Nephroprotective activity

Bioactive flavonoids, isolated from *B. rotunda*, manifest a range of pharmacological properties, notably the ability to avert kidney injury, thereby positioning them as traditional nephroprotective agents. In the investigation conducted by Sujana et al. (2024), the nephroprotective efficacy of the ethanolic extract of *B. rotunda* rhizome (EEBR) was assessed across dosages of 125, 250, and 500 mg/kg BW. Comparative analysis with the negative control group revealed a significant reduction in serum creatinine, urea nitrogen, glutamic pyruvic transaminase, and malondialdehyde levels. Furthermore, the cisplatin-induced upregulation of renal cortex genes, including kidney injury molecule 1 (KIM-1), neutrophil gelatinase-associated lipocalin (NGAL), caspase 3, and caspase 7, in rats was substantially mitigated. In parallel, EEBR exerted no significant influence on body weight or the relative kidney weight percentage, thus bolstering its safety profile. Consistent with these findings, the natural flavonoid Pinostrobin (**1**), derived from *B. rotunda*, has been shown by Ijaz et al. (2023) to possess the ability to ameliorate renal injury in rats. Pinostrobin (**1**) was demonstrated to significantly mitigate cadmium-induced renal impairment by modulating cadmium-induced alterations in urea, creatinine, anti-oxidant enzyme activities, creatinine clearance, circulating enzymes of the tricarboxylic acid cycle, renal oxidative stress, apoptosis, and inflammation. These renoprotective effects are hypothesized to be mediated through Pinostrobin’s (**1**) anti-inflammatory, anti-apoptotic, and anti-oxidant properties. In a parallel study, Worakajit et al. (2022) explored the protective effects of Panduratin A (**20**), an active metabolite derived from *B. rotunda*, against mucin-induced nephrotoxicity in rats using both *in vivo* and *in vitro* methodologies. The experimental outcomes indicated that treatment with 2.5 and 25 mg/kg BW Panduratin A (**20**) effectively attenuated the increase in blood urea nitrogen (BUN) following visfatin administration, inhibited visfatin-induced apoptosis in renal tubular epithelial cells, and neutralized the mitochondrial damage provoked by visfatin in these cells. Additionally, Panduratin A (**20**) led to a downregulation in the expression of carbonyl proteins, cytochrome c, caspase 3, T cell immunoglobulin domain and mucin domain protein-1 (Tim-1), and NGAL, while concurrently upregulating the expression of Bcl-2 within renal tissues and renal proximal tubular cells. Thongnuanjan et al. (2021) employed the *in vitro* cultured human renal cell line RPTEC/TERT 1 to investigate the impact of Panduratin A (**20**) on cisplatin-induced toxicity. Their findings indicated that Panduratin A (**20**) ameliorates cisplatin-induced nephrotoxicity in both mice and RPTEC/TERT 1 cells by diminishing apoptotic cell death. The oral administration of Panduratin A (**20**) at a dosage of 50 mg/kg BW was found to enhance renal function and alleviate cisplatin-induced renal tubular damage. This salutary effect is believed to be mediated through the inhibition of the extracellular signal-regulated kinase (ERK) 1/2 and caspase-3 signaling pathways.

In summary, *B. rotunda* exhibits a significant capacity to safeguard renal health through the modulation of multiple renal function pathways. The plant has been shown to effectively lower blood levels of creatinine, urea nitrogen, glutamate pyruvate transaminase, and malondialdehyde, which suggests its considerable therapeutic potential in the treatment and prevention of kidney injury. Nonetheless, the precise mechanisms underlying the nephroprotective activity of *B. rotunda* extracts and metabolites remain partially elucidated, with several studies focusing primarily on pharmacodynamic assessments rather than delving into specific pathological mechanisms. Follow-up studies should further analyze how *B. rotunda* exerts its nephroprotective activity through *in vivo* and *in vitro* experiments, so as to explore the mechanism of the nephroprotective effect of *B. rotunda*. In addition, further toxicological and clinical studies are necessary to convert the remarkable activity of *B. rotunda* into clinical applications.

### 6.3 Anti-inflammatory activity

In their 2022 investigation, Jamornwan et al. (2022) evaluated the anti-inflammatory effects of Panduratin A (**20**), a metabolite isolated from *B*. *rotunda*, on lipopolysaccharide (LPS)-induced microglia activation in the spontaneous immune microglia (SIMA9) model. The study revealed that Panduratin A (**20**) displayed potent biological activity across a range of concentrations (0.1, 1, 5, 10, and 50 μM), reducing the levels of nitric oxide (NO) and p-NF-κB p65 in a concentration-dependent manner. Moreover, Panduratin A (**20**) significantly diminished the secretion of pro-inflammatory cytokines, including tumor necrosis factor-α (TNF-α), interleukin-1β (IL-1β), and interleukin-6 (IL-6), while markedly enhancing the release of anti-inflammatory cytokines, interleukin-4 (IL-4) and interleukin-10 (IL-10), from LPS-stimulated cells. Consequently, this resulted in an increased production and secretion of anti-inflammatory mediators, highlighting the immunomodulatory potential of Panduratin A (**20**) in the context of neuroinflammation. Mohan et al. (2020) studied the gastric protective effect of Boesenbergin A (**35**) isolated from the extract of *B. rotunda* by using ethanol-induced gastric ulcer model in rats. The tissue malondialdehyde (MDA) levels were significantly decreased by 14.01 and 7.13 μmol/g under the pretreatment of 10 and 20 mg/kg BW doses, respectively. In addition, Boesenbergin A (**35**) significantly restored elevated prostaglandin E2 (PGE2) levels while reducing NO and inducible NO synthase (iNOS) levels. Rosdianto et al. (2020) conducted a study to elucidate the anti-inflammatory mechanisms of the ethanol extract derived from *B. rotunda*. Their research demonstrated that the extract significantly reduced the infiltration of inflammatory cells in the gastric and intestinal tissues of rats subjected to acetic acid-induced inflammation. Additionally, the extract modulated the expression levels of key regulatory proteins, Akt and NF-κB p65. These findings provide a scientific basis for the potential application of *B. rotunda* extract in anti-inflammatory therapy and gastric protection, underscoring its therapeutic potential in mitigating inflammatory responses and safeguarding gastrointestinal health.

Pui Ping et al. (2020) explored the protective effects of Cardamonin (**27**), a metabolite derived from *B. rotunda*, against inflammation-mediated pain and injury. Their findings imply that the injury-preventive properties of Cardamonin (**27**) may be associated with its capacity to modulate protein kinase C (PKC) activity, N-methyl-D-aspartic acid (NMDA) and non-NMDA glutamate receptor function, the L-arginine/nitric oxide/cGMP signaling pathway, and ATP-sensitive potassium channels. Notably, Cardamonin (**27**) significantly suppressed acetic acid-induced pain responses in a dose-dependent manner at concentrations of 0.3, 1, 3, and 10 mg/kg BW, with corresponding inhibition rates of 45%, 56%, 80%, and 100%, respectively. These results suggest that the anti-inflammatory and analgesic effects of Cardamonin (27) may be attributed to its influence on PKC activity, glutamate receptor activity, and associated signaling pathways. In a comparable study, Ping et al. (2018) observed a significant dose-dependent inhibition of acetic acid-induced abdominal torsional pain following both intraperitoneal injection and oral administration of Cardamonin (**27**) at doses of 0.3, 1, 3, and 10 mg/kg BW. Concurrently, Cardamonin (27) demonstrated notable analgesic effects in models induced by formalin, capsaicin, and glutamate. However, the precise mechanisms underlying these anti-inflammatory and analgesic effects of Cardamonin (**27**) require further elucidation. Voon et al. (2017) conducted a study to investigate the pharmacological activity of Cardamonin (**27**) in a rheumatoid arthritis model, which was established by plantar injection of complete Freund’s adjuvant in Sprague-Dawley rats. Treatment with varying concentrations of Cardamonin (**27**), specifically 0.625, 1.25, and 2.5 mg/kg BW, resulted in a significant reduction in plasma concentrations of IL-6 and IL-1β. Notably, only the lowest dose of 0.625 mg/kg BW of Cardamonin (**27**) elicited a significant decrease in plasma TNF-α levels within the rheumatoid arthritis model. These results highlight the capacity of Cardamonin (**27**) to substantially suppress complete Freund’s adjuvant-induced inflammatory changes in the joints of rats with rheumatoid arthritis.

Furthermore, Boonyarattanasoonthorn et al. (2023) conducted a pharmacokinetic study of Panduratin A (**20**) in Beagle dogs, thereby establishing its oral safety and laying a scientific foundation for the potential use of botanical drugs based on *B*. *rotunda* extract in the context of COVID-19 pandemic management. In a related study, Kongratanapasert et al. (2023) reported that Panduratin A (**20**), extracted from *B. rotunda*, led to significant decreases in lung pathophysiology and inflammatory mediators, including PGE2 and IL-6, in hamsters infected with SARS-CoV-2. Notably, PA showed high bioavailability at dosages of 300 and 1,000 mg/kg BW. In a similar vein, Kanjanasirirat et al. (2020) demonstrated the potent anti-SARS-CoV-2 activity of Panduratin A (**20**) in Vero E6 cells, with an IC_50_ value of 3.62 μg/mL (CC_50_ = 28.06 μg/mL) and 0.81 μM (CC_50_ = 14.71 μM), indicating its potential as an anti-viral agent. These findings suggest that Panduratin A (**20**) possesses not only oral safety and high bioavailability but also exhibits potential dual efficacy as an anti-inflammatory and anti-viral agent. This dual functionality renders Panduratin A (**20**) a promising candidate for the therapeutic management of COVID-19 and associated inflammatory conditions.

In aggregate, a plethora of experimental studies have demonstrated a strong correlation between the anti-inflammatory activity of *B. rotunda* and its abundant flavonoid content. Notably, flavonoids such as panduratin A (**20**) have been proven to exert significant anti-inflammatory effects by inhibiting pivotal inflammatory mediators, including NO, PGE2, and TNF-α. Despite these findings, the extant research on the anti-inflammatory activities of *B. rotunda* is not sufficiently comprehensive to elucidate the underlying mechanisms fully. Consequently, there is a clear imperative for subsequent, more profound investigations into the anti-inflammatory mechanisms of *B. rotunda*. Moreover, flavonoids have garnered considerable interest due to their potential in drug discovery. The precise modification of these flavonoids through chemical synthesis or targeted molecular approaches could enhance their therapeutic precision and efficacy. Such advancements could pave the way for the development of novel pharmaceuticals with augmented potency and selectivity. Therefore, it is essential to delve deeper into the study of flavonoids in *B. rotunda* to harness their full therapeutic potential and contribute significantly to medical advancement.

### 6.4 Anti-bacterial activity

The anti-bacterial and anti-viral properties of *B. rotunda* have been well-documented in literature. In a particular study, Thadtapong et al. (2024) delineated the potency of a novel flavonoid, extracted from *B. rotunda*, in impeding biofilm formation of two notable strains, Aci46 and Aci44. Additionally, they illustrated the capacity of panduratin A (**20**), at concentrations of 2.5 and 5 μM, to potentiate the activity of colistin. This revelation underscores the potential of panduratin A (**20**) to function as an adjuvant for mucilage, augmenting the efficacy of existing anti-microbial agents. More recently, Apinundecha et al. (2023) explored the combined anti-microbial effects of *B. rotunda* essential oil (BREO) and cloxacillin. Their findings indicated that BREO possessed notable anti-bacterial properties, capable of decreasing the activity of *Staphylococcus aureus*, with a minimum inhibitory concentration (MIC) of 2 mg/mL.

Biofilm formation is a key virulence factor associated with the opportunistic fungal pathogen *Candida albicans*. The principal metabolites of *B. rotunda* extract, Pinostrobin (**1**) and Pinocembrin (**2**), have been shown to substantially inhibit biofilm formation. Kanchanapiboon et al. (2020) reported that both the extract and Pinocembrin (**2**) significantly reduced cell surface hydrophobicity and downregulated ALS3 mRNA expression levels. The extract exerted a significant inhibitory effect on biofilm development, with an IC_50_ value of 17.7 μg/mL, indicating its potency. In parallel, *B. rotunda* extract displayed considerable inhibitory activity against beta-lactam-resistant strains of *S. aureus* and *Staphylococcus epidermidis*. Teethaisong et al. (2018) elucidated the mechanism of action, which involves interaction with the bacterial cytoplasmic membrane, leading to membrane disruption and subsequent leakage of intracellular contents. Blastocystis hominis, a protozoan parasite, can cause a spectrum of gastrointestinal symptoms in humans and animals. Kaewjai et al. (2023) determined that at a MIC of 62.5 μg/mL, *B. rotunda* extract significantly suppressed the growth of *Blastocystis hominis*, inducing cellular crumpling and exhibiting significant anti-protozoal activity.

To date, the majority of research has concentrated on the potent *in vitro* anti-bacterial effects of *B. rotunda*, with a relative scarcity of *in vivo* studies. The adverse reactions and potential for drug resistance associated with *B. rotunda* remain undetermined, and an accurate assessment of drug interactions, their subsequent reactions, and effects within biological systems is yet to be fully realized. It is essential to further investigate the *in vivo* responses, anti-bacterial mechanisms, and physiological effects of *B. rotunda* to gain a comprehensive understanding of its therapeutic potential and biological interactions. This includes examining how *B. rotunda* not only inhibits or eradicates certain bacteria but also modulates immune responses, thereby enhancing anti-bacterial efficacy through various mechanisms, the precise role of which remains to be elucidated. Consequently, an in-depth study of the *in vivo* responses, anti-microbial mechanisms and physiological effects of *B. rotunda* is essential for a comprehensive understanding of its therapeutic potential and biological interactions.

### 6.5 Anti-obesity activity

Obesity is a critical factor in the development of chronic metabolic syndromes. The metabolites Panduratin A (**20**), Cardamonin (**27**), and Isopanduratin A (**21**), all of which are derived from *B. rotunda*, have been identified as potent anti-obesity agents. These metabolites demonstrate both anti-adipogenic and lipolytic activities, highlighting their potential in the management of obesity-related metabolic disorders. Rungsa et al. (2023) conducted a study revealing that isopanduratin A (**21**), a metabolite of *B. rotunda*, potently inhibited adipogenesis by specifically targeting the mitogen-activated protein kinase (MAPK) pathway and modulating the AKT/GSK3β and AKT/AMPK-ACC signaling cascades. Notably, isopanduratin A (**21**) impeded ERK phosphorylation during the initial phase of adipogenesis, thereby suppressing mitotic clonal expansion. This distinctive attribute has not been observed for Pinostrobin (**1**) and, to date, has not been reported for Panduratin A (**20**) or Cardamonin (**27**), underscoring the unique potential of isopanduratin A (**21**) in the context of anti-obesity therapeutics. San et al. (2022) conducted a study demonstrating that Panduratin A (**20**), at concentrations above 5 μM, significantly reduced both cell viability and triglyceride levels. Notably, Panduratin A (**20**) did not affect mitotic clonal expansion during adipogenesis. At concentrations between 5 and 20 μM, Panduratin A (**20**) markedly decreased the mRNA expression levels of key adipogenic factors CCAAT/enhancer-binding protein α (C/EBPα**)**, peroxisome proliferator-activated receptor γ (PPARγ), and sterol regulatory element binding protein-1c (SREBP-1c), along with the protein expression of phosphorylated protein kinase B (p-Akt), phosphorylated c-jun N-terminal kinase (p-JNK), and phosphorylated p38 mitogen-activated protein kinase (p-p38). Compared to the control group, a significant reduction in the p-JNK/JNK ratio was observed at the lower concentration of 5 µM of Panduratin A (**20**), while the suppression of the p-p38/p38 ratio was evident at higher concentrations. The accumulation of cellular lipid droplets, as assessed by the oil red O staining assay, showed reductions to 92.3%, 85.4%, and 51.2% following treatment with 5, 10, and 20 µM of Panduratin A (20), respectively. This indicates a substantial decrease in lipid droplet accumulation in cells treated with Panduratin A (**20**). In a related study by Jitsaeng et al. (2023), Panduratin A (20), isolated from fraction 3 (BP-3) of the ethanolic extract of *B. rotunda*, was found to exhibit anti-adipogenic and lipolytic activities. In their study, the BP-3 extract, containing 0.29 g/g extract of Panduratin A (**20**), was non-toxic to cells and demonstrated more potent anti-adipogenic and lipolytic effects on 3T3-L1 preadipocyte cells at a concentration of 10 μg/mL. Furthermore, Chatsumpun et al. (2017) characterized a range of metabolites in *B. rotunda* extract that exhibit inhibitory activities against α-glucosidase and pancreatic lipase, with biflavones and chalcones being the predominant metabolites. Potipiranun et al. (2018) observed the inhibition of glycosylation and α-glucosidase by metabolites derived from *B. rotunda*. From the dichloromethane extract, three flavanones, two chalcones, two dihydrochalcones, and one narcotic piperolactone were isolated. These metabolites demonstrated more potent inhibition of advanced glycation end products compared to aminoguanidine. It was also observed that activity varied among different structural types. Metabolites featuring hydroxyl and α-β unsaturated ketone structures showed greater activity, whereas metabolites with methoxy and geranyl groups exhibited reduced activity. Notably, the presence of a methoxy group at the C-4 position of dihydrochalcone was more effective than that at the C-6 position. Additionally, Pinocembrin (**2**) showed moderate inhibitory effects against maltase and sucrose, with IC_50_ values of 0.35 mM and 0.39 mM, respectively. These findings underscore the structure-activity relationships among the metabolites of *B. rotunda* and their potential implications in the management of diabetes and obesity.

*B. rotunda* has been shown to have a significant ability to inhibit lipogenesis, suggesting that it could be used in foods and drugs for the prevention and control of obesity. However, current studies are mainly limited to a few metabolites and further exploration of other metabolites or extracts is scarce. Meanwhile, the identified lipogenesis inhibitory activities have not been thoroughly investigated and detailed mechanistic studies are lacking, thus preventing a precise assessment of the anti-obesity mechanism of Verticillium rolfsii. Therefore, it is important to thoroughly grasp the mechanism of action of *B. rotunda* and comprehensively assess the therapeutic potential of *B. rotunda*, thereby laying a solid foundation for safe and effective translation into clinical practice.

### 6.6 Hepatoprotective activity

The hepatoprotective effects of the chalcone Panduratin A (**20**) were assessed in a rat model of liver injury induced by intraperitoneal administration of thioacetamide (TAA). In a study conducted by Salama et al. (2018), the protective effects of *B. rotunda* extracts on liver cirrhosis were investigated. The findings revealed that Panduratin A (**20**) substantially decreased the severity of liver injury, activation of hepatic stellate cells (HSCs), and collagen deposition. Furthermore, Panduratin A (**20**) significantly elevated the levels of superoxide dismutase (SOD), catalase (CAT), and glutathione peroxidase (GPx), thereby establishing its hepatoprotective mechanism through the inhibition of HSC activity. In a distinct study, Salama et al. (2013) established a liver injury model in SD rats by intraperitoneal injection of 200 mg/kg BW TAA. The research demonstrated that the oral administration of *B. rotunda* extract exerts a significant protective effect against liver injury in rats. Histopathological examination of the rat livers revealed marked amelioration following *B. rotunda* extract intervention. Furthermore, *B. rotunda* extract was found to reduce the levels of liver nitrotyrosine and urine 8-OH-dG, enhance the activities of CAT and GPx, and promote hepatocyte survival. The primary mechanisms by which *B. rotunda* extract ameliorates liver injury in rats encompass the inhibition of fibrotic factors such as transforming growth factor beta 1 (TGF-β1), NF-κB, and IL-6, upregulation of caspase-1 expression, elevation of serum levels of Bax and caspase-3, and suppression of extracellular matrix proteins and hepatocyte proliferation.

The hepatoprotective significance of *B. rotunda* is increasingly acknowledged, particularly for its ability to curb the proliferation of fibrotic cells and to trigger apoptosis. Despite this, research dedicated to harnessing its potential in the prevention and treatment of liver injury remains sparse, with a notable absence of mechanistic studies. Considering the multifaceted origins of hepatocellular injury, the mechanisms and pathologies are highly heterogeneous, and thus further refinement of its therapeutic mechanism of action is needed. A comprehensive understanding of the therapeutic mechanisms of *B. rotunda* is essential to effectively translate the potential of *B. rotunda* into clinical practice and to ensure the safety and efficacy of liver disease treatment.

### 6.7 Anti-oxidant activity

The anti-oxidant potency of chalcone Panduratin A (**20**) was assessed by Salama et al. (2013) using 2,2-Diphenyl-1-picrylhydrazyl (DPPH) free radicals, ferric reducing power, and a normal embryonic cell line WRL-68. The experimental results demonstrated that Panduratin A (**20**) harbors anti-oxidant properties, with a dose-dependent decrease in DPPH radical content and an increase in iron-reducing power activity. Additionally, it was observed that Panduratin A (**20**) significantly reduced MDA levels and bolstered the activities of SOD, CAT, and GPx, thereby further enhancing cellular anti-oxidant defenses. Abdelwahab et al. (2011) conducted a study on the methanol extract of *B rotunda* and its principal metabolite, Pinostrobin (**1**), to evaluate their anti-ulcer and anti-oxidant activities against ethanol-induced gastric ulcers. The experimental results indicated that the extract offered protection to the gastric mucosa, as evidenced by a reduction in ulcer area and mucosal damage, along with the mitigation or resolution of submucosal edema and leukocyte infiltration. Additionally, *in vitro* assessments revealed that the extract significantly suppressed the production of NO by LPS/IFN-γ-activated rodent cells, while showing no inhibitory effect on cyclooxygenase-1 (COX-1) and cyclooxygenase-2 (COX-2). In a subsequent study, Isa et al. (2012) assessed the anti-oxidant activity of *B. rotunda* using the oxygen radical absorbance capacity assay, with Quercetin (**6**) serving as a standard anti-oxidant reference. Both *B. rotunda* at a concentration of 20 μg/mL and Quercetin (**6**) at 5 μg/mL, equivalent to 11.91 and 160.32 µM Trolox concentrations, respectively, exhibited robust anti-oxidant activity by inhibiting the COX-2 enzyme.

At present, research on *B. rotunda* has demonstrated promising anti-oxidant activity, garnering significant interest within the scientific community due to its implications for therapeutic applications. Nonetheless, the current body of research is limited, particularly with regard to *in vivo* studies, and there is a pressing need for more comprehensive investigations to elucidate the mechanisms underlying the anti-oxidant effects of its metabolites. These studies are crucial for advancing our comprehension of *B. rotunda*’s pharmacological activity and for optimizing its utilization in the development of pharmaceuticals.

### 6.8 Other bioactivities

Pinostrobin (**1**), a naturally occurring bioflavonoid metabolite derived from the rhizomes of *B. rotunda*, has been the subject of scientific scrutiny. In a study conducted by Kongsui et al. (2023), Parkinson’s disease rat models were administered a dosage of 40 mg/kg BW Pinostrobin (**1**), which resulted in a marked reduction of free radicals within the substantia nigra, a significant decrease in MDA levels, and a substantial increase in anti-oxidant enzyme activities, namely, SOD and GSH. These biochemical changes corresponded with an improvement in the locomotor function of the rats, indicative of a therapeutic effect. In a parallel investigation, Thongrong et al. (2023) determined that Pinostrobin (**1**) mitigated chronic stress-induced cognitive deficits and averted brain dysfunction by exhibiting anti-oxidant properties that diminished neuronal cell damage and bolstered the functionality of astrocyte markers, glial fibrillary acidic protein (GFAP), and excitatory amino acid transporter 2 (EAAT2). Pinostrobin (1), a bioflavonoid metabolite isolated from *B. rotunda*, has demonstrated anti-oxidant properties and the capacity to facilitate the repair of peripheral nerve damage. In a study by Kongsui et al. (2023), male Wistar rats with sciatic nerve crush injuries were subjected to treatment with Pinostrobin (**1**) at dosages of 20 and 40 mg/kg BW to assess its reparative effects. The findings indicated that Pinostrobin (**1**) treatment at both concentrations mitigated oxidative stress by increasing endogenous glutathione levels. This upregulation led to an enlargement of axon diameter, an increase in the count of dorsal root ganglion (DRG) neurons, and an enhanced expression of phosphorylated extracellular signal-regulated kinase 1/2, thereby promoting the recovery of sciatic nerve functionality. Collectively, the study suggests that Pinostrobin (**1**) could serve as an effective therapeutic strategy for the treatment of peripheral nerve injuries. Furthermore, Wang et al. (2022) reported in their study that the *B. rotunda* extract possesses anti-inflammatory and analgesic effects. Diabetic peripheral neuropathy was induced in SD rats by administering a combination of 30% fructose solution and streptozotocin. Throughout the experiment, the administration of *B. rotunda* extract at dosages of 100 and 400 mg/kg BW significantly reduced blood glucose levels and attenuated weight loss in diabetic rats, demonstrating a hypoglycemic effect. Additionally, the analysis of serum pro-inflammatory cytokines revealed that the concentrations of TNF-α and IL-1β in the serum of diabetic rats treated with *B. rotunda* extract were significantly lower compared to the normal control group, with the reduction exhibiting a dose-dependent pattern.

Targeting angiogenesis represents a compelling and promising therapeutic strategy in oncology. Adhikari et al. (2020) revealed that *B. rotunda* extract elicited relaxation in coronary artery rings via both endothelium-dependent mechanisms, such as the nitric oxide-cGMP pathway, and endothelium-independent mechanisms, including the blockade of Ca^2+^ channels. Furthermore, flavonoids with vasorelaxant activity were isolated and characterized from the active fractions, comprising Naringenin 5-methyl ether (**5**), Alpinetin (**3**), pinocembrin (**2**), Pinostrobin (**1**), and 4-hydroxypanduratin A (**22**). These metabolites all demonstrated significant vasodilatory effects, although the precise mechanisms responsible for these actions remain to be fully elucidated. Additionally, Bereswill et al. (2012) investigated the anti-angiogenic properties of the natural chalcone Panduratin A (**20**), originating from *B. rotunda*. This was substantiated through both *in vitro* and *in vivo* studies. *In vitro* evaluations indicated that Panduratin A (**20**) displayed concentration-dependent cytotoxicity and growth inhibition in human umbilical vein endothelial cells, distinct from its effects on normal human fibroblasts and liver epithelial cells, with an IC_50_ of 6.91 mM. *In vivo*, the inhibitory effect of Panduratin A (**20**) on neointimal formation was confirmed using the mouse Matrigel plug assay and the zebrafish angiogenesis model, thereby highlighting its potential as an anti-angiogenic agent. Ruttanapattanakul et al. (2022) characterized Pinocembrin (**2**) as an active metabolite exerting potent stimulatory effects on human keratinocytes. Following a 15 min exposure to Pinocembrin (2) at concentrations of 15.6, 31.3, and 62.5 μM, the activation of two key kinases, ERK1/2 and Akt, was rapidly triggered in the individual cells. This finding underscores the potential utility of plants and natural products rich in Pinocembrin (**2**) as viable alternatives for skin regeneration and wound healing therapies.

In summary, *B. rotunda* exhibits potential in mitigating heat-induced pain, cold-induced pain, and mechanical allodynia in diabetic rats, while concurrently enhancing motor coordination through its anti-oxidant properties, reducing neuronal damage, and upregulating astrocyte marker functions such as GFAP and EAAT2. Furthermore, it demonstrates anti-cancer activity by suppressing cancerous lesions and promoting overall bodily function, with a particular impact on angiogenesis. As a result, *B. rotunda* presents itself as a promising candidate for use as an adjunct therapy or a source of pharmaceutical leads in the therapeutic management of a spectrum of conditions, including Alzheimer’s disease, Parkinson’s disease, neurotraumatic injuries, and neoplastic diseases. Subsequently, a thorough investigation of these activities is warranted to elucidate their underlying mechanisms of action, thereby contributing to advancements in the field of medicine.

## 7 Quality control

The quality standardization of medicinal products is designed to ensure the consistency of quality and composition in traditional Chinese medicine, thereby ensuring the reliability of its clinical efficacy (Wu et al., 2018; Wang et al., 2024). Currently, the analysis of active metabolites in *B. rotunda* primarily focuses on flavonoids. *B. rotunda* is rich in a variety of flavonoids. Ruttanapattanakul et al. (2021) employed high-performance liquid chromatography to analyze the metabolite profile of various *B. rotunda* extracts, identifying kaempferol (**4**) as a predominant bioactive metabolite. Their methodology demonstrated precision and stability in quantifying kaempferol content. Furthermore, complementary chromatographic analyses of the ethyl acetate fraction of *B. rotunda* by other researchers revealed eight flavonoids, which are considered the signature chemical metabolites characteristic of *B. rotunda* (Adhikari et al., 2020; Kanchanapiboon et al., 2020) determined the chemical metabolites of the extract by UPLC. Two flavonoids, Pinostrobin (**1**) and Pinocembrin (**2**), were determined from the extract. The contents of the two metabolites were 25.2% and 11.6%, respectively. Another study utilized LC-MS to quantify Quercetin (**6**) in the ethanol extract of *B. rotunda* rhizomes, establishing a method for its determination and confirming the presence of Quercetin (**6**) (Rosdianto et al., 2020). Additionally, Yuliana et al. (2013) utilized a comprehensive extraction-nuclear magnetic resonance metabolomics approach to identify adenosine A1 receptor-binding metabolites in the rhizome extract of *B. rotunda*. Their study led to the discovery of two flavonoids, pinocembrin (**2**) and hydroxy-panduratin (**49**). This methodology facilitates the expedited identification of bioactive metabolites from plant extracts, circumventing the requirement for prior purification.

From a biological perspective, the identification of *B. rotunda* has emerged as a focal point of new research, offering a fresh direction in its characterization. Researchers applied PCR technology to conduct an exhaustive examination of the entire chloroplast genome of *B. rotunda*. Notably, within the large single-copy (LSC) and small single-copy (SSC) regions of the chloroplast genome, they discovered multiple highly polymorphic regions, such as psbK-psbI, trnT-GGU-psbD, rbcL-accD, ndhF-rpl32, and ycf1. Due to their high polymorphism, these regions can serve as molecular markers for DNA barcoding and species delineation, thereby providing a crucial reference for the identification of *B. rotunda* (Liew et al., 2022).

The establishment and enforcement of quality standards for *B. rotunda* are instrumental in advancing the standardization of production and distribution processes, thereby fostering the robust growth of the traditional Chinese medicine sector. Given its dual role as both a medicinal and edible resource, the formulation of stringent quality benchmarks is of paramount importance. At present, the quality standards for *B. rotunda* predominantly concentrate on flavonoid content, with research into quality criteria for other metabolites remaining comparatively scarce. Consequently, there is a critical need to augment and refine the quality assessment parameters to ensure comprehensive quality assurance for *B. rotunda*.

## 8 Pharmacokinetics

Pharmacokinetics is dedicated to elucidating the dynamic processes of drug disposition within the human body, providing a crucial scientific foundation for the realization of personalized therapeutic regimens, as well as for ensuring the efficacy and safety of drug treatments (Li et al., 2020; Feng et al., 2024). Boonyarattanasoonthorn et al. (2023) investigated the pharmacokinetic profile of Panduratin A (**20**) in beagles. They found no statistically significant differences between two pharmacokinetic parameters following intravenous administration of 1 mg/kg BW Panduratin A (**20**) and multiple oral doses of 5 and 10 mg/kg BW of *B. rotunda* extract formulations. Whereas, C_max_ and area under the curve (AUC) of the *B. rotunda* extract preparations were positively correlated with the dose of the oral drug. The conversion of Panduratin A (**20**) to other metabolites primarily occurs through oxidative and glucuronidation pathways, while its elimination from the body mainly occurs via the fecal route. This indicates the safety of both intravenous administration of the pure metabolite and oral administration of *B. rotunda* extracts. The findings of the study indicate that the pharmacokinetic profile of Panduratin A (**20**) closely resembles that of orally administered *B. rotunda* extracts. Certain researchers utilized LC-MS to quantify the levels of Panduratin A (**20**) in the plasma and lungs of hamsters infected with SARS-CoV-2 after treatment with *B. rotunda* extracts. They observed a dose-dependent increase in its levels. Specifically, at a dosage of 1,000 mg/kg BW, the concentration of Panduratin A (**20**) attained values ranging from 7,000 to 9,000 μg/L in plasma and 1,500–2,500 μg/L in lung tissues, leading to a significant reduction in lung inflammation and cytokine levels. However, pharmacokinetic studies of *B. rotunda* remain quite limited.

In conclusion, the pharmacokinetic profile of *B. rotunda* is crucial for the development and optimization of its pharmaceutical applications. From the results of the current study, it was found that there are still inherent limitations, fewer pharmacokinetic studies and lack of systematic pharmacokinetic studies. Future studies should focus on elucidating the absorption kinetics of the active metabolites in *B. rotunda*, including the rate and extent of absorption. It is also important to explore the distribution characteristics of these metabolites *in vivo*, to assess their concentrations in different tissues and organs, and to determine whether there is a specific targeting tendency. As well as studies on the metabolic and excretory pathways of the active metabolites and their derivatives. These will greatly contribute to the clinical application of *B. rotunda* and provide some help to the drug research of *B. rotunda*.

## 9 Toxicity assessment

As previously noted, *B. rotunda* possesses a venerable history of utilization in China, serving as a valued and reputedly safe traditional Chinese medicine as well as a ubiquitous dietary staple. While the literature on the toxicity and safety assessment of *B. rotunda* is somewhat scarce, ongoing surveillance of its usage is warranted. Notably, Panduratin A (**20**) and Pinostrobin (**1**), metabolites isolated from *B. rotunda*, have been documented to exhibit no clinical signs of toxicity, morbidity, or mortality following continuous administration over a 90-day period at dosages of 25, 50, or 100 mg/kg BW (Techapichetvanich et al., 2024). Saah et al. (2024) employed freeze-drying technology to encapsulate *B. rotunda* extract and investigated the impact of maltodextrin (MD), arabic gum (GA), and their blends (MDGA) with various wall materials on the biological activity of the encapsulated product. The study demonstrated that the incorporation of MDGA as a carrier material mitigated the cytotoxicity associated with MDGA-5 and MDGA-7 formulations, concurrently enhancing the viability of MC3T3-E1 cells and the activity of alkaline phosphatase. These findings suggest that the MDGA wall material not only augments the biological efficacy of the *B. rotunda* extract but also contributes to the preservation of the stability of its bioactive metabolites. Consequently, this encapsulation technique is projected to be instrumental in the development of therapeutic and nutritional products designed for the management or prevention of osteoporosis. Ruttanapattanakul et al. (2022) performed cytotoxicity assays of Pinocembrin (**2**) in immortalised human keratinogenic HaCaT cells and showed that Pinocembrin (**2**) between 15.6 and 125 µM presented a significant increase in cell viability/cell proliferation. However, 500 µM was the only Pinocembrin (**2**) concentration that significantly reduced HaCaT cell viability. In addition, Panduratin A (**20**), isolated from the ethanol extract fraction 3 (BP-3) of *B. rotunda*, was further studied. The findings revealed that the BP-3 extract contained a notably high amount of Panduratin A (**20**). At concentrations below 10 μg/mL, it exhibited no cytotoxicity towards 3T3-L1 cells, yet demonstrated potent anti-lipogenic and lipolytic activities at this same concentration range (Jitsaeng et al., 2023).

Most current studies primarily focus on short-term effects, with limited investigation into long-term chronic toxicity. Additionally, there is a notable absence of comprehensive assessments of organ-specific toxic effects, which are essential for understanding potential side effects in clinical use. Furthermore, more detailed dose-response studies are necessary to precisely determine the threshold between therapeutic and toxic doses. Future research should include acute, subacute, and chronic toxicity studies to fully understand the safety profile of *B. rotunda*. Establishing a standardized toxicity test protocol will ensure consistency and comparability across various studies. Additionally, exploring the potential synergistic toxic effects of *B. rotunda* when combined with other drugs or supplements is crucial. Moreover, long-term clinical trials should be initiated to evaluate the safety and efficacy of *B. rotunda* in real-world settings, providing valuable data for its clinical application (Wang et al., 2024). To explore the effects of long-term exposure to different dose levels, and to develop better predictive models to evaluate the safety factor. Such a systematic investigation will provide a solid scientific basis for the safe and effective utilization of *B. rotunda* in clinical settings.

## 10 Clinical settings

At present, although it is found that *B. rotunda* has good biological activity *in vitro* and *in vivo*, there is a lack of certain clinical data, and there are relatively few related clinical studies. This situation limits our comprehensive understanding of the efficacy and safety of *B. rotunda*. Jitsaeng et al. (2023) selected 13 healthy volunteers to investigate the anti-lipogenesis and lipolysis effects of Panduratin A (**20**) microneedle serum. The volunteers applied the microneedle serum containing Panduratin A (**20**) to their thigh skin and gently massaged the area for 14 days. The results indicated a decrease in thigh circumference, along with increased skin hydration and firmness after treatment, suggesting its potential as a lipolysis agent. In addition, a single-blind, randomized, cross-over study evaluated the preventive and alleviating effects of a traditional Chinese medicine mouthwash containing *B. rotunda* extract on radiation-induced oral mucositis. A total of 120 patients undergoing radiotherapy for head and neck cancer were randomly divided into three treatment groups receiving normal saline, sodium bicarbonate, or the traditional Chinese medicine extract gargle. The therapeutic effects were assessed using mucositis score and body mass index. The results showed that the traditional Chinese medicine mouthwash could slightly delay the onset of symptoms, with an average mucositis score of less than two across all groups and no significant difference in body mass index. These findings suggest that the traditional Chinese medicine gargle may serve as an effective alternative to bicarbonate and normal saline for the preventive treatment of radiation-induced oral mucositis (Kongwattanakul et al., 2022). Chitapanarux et al. (2021) conducted a 4-week randomized, double-blind, placebo-controlled study to assess the effectiveness and safety of *B. rotunda* extract in individuals with functional dyspepsia. The outcomes indicated that patients receiving *B. rotunda* extract experienced significant alleviation in symptoms such as dyspepsia, heartburn, and reflux. Furthermore, the treatment group demonstrated superior overall symptom improvement compared to the placebo group. In addition, the level of blood inflammatory markers in the treatment group was reduced, showing an inhibitory effect on gastric body inflammation. These findings suggest that *B. rotunda* extract may be an effective method for the treatment of functional dyspepsia.

The above studies have provided valuable insights into the pharmacological effects of *B. rotunda* in humans, thereby expanding the range of clinical therapeutic options. Although modern pharmacological studies have demonstrated that *B. rotunda* possesses a wide range of biological activities, they have failed to translate into clinical applications, and the scarcity of clinical trials has limited the full realization of *B. rotunda*’s potential. Therefore, there is an urgent need to further investigate the multiple pharmacological activities of *B. rotunda* in clinical practice.

## 11 Bibliometrics

**FIGURE 8:**
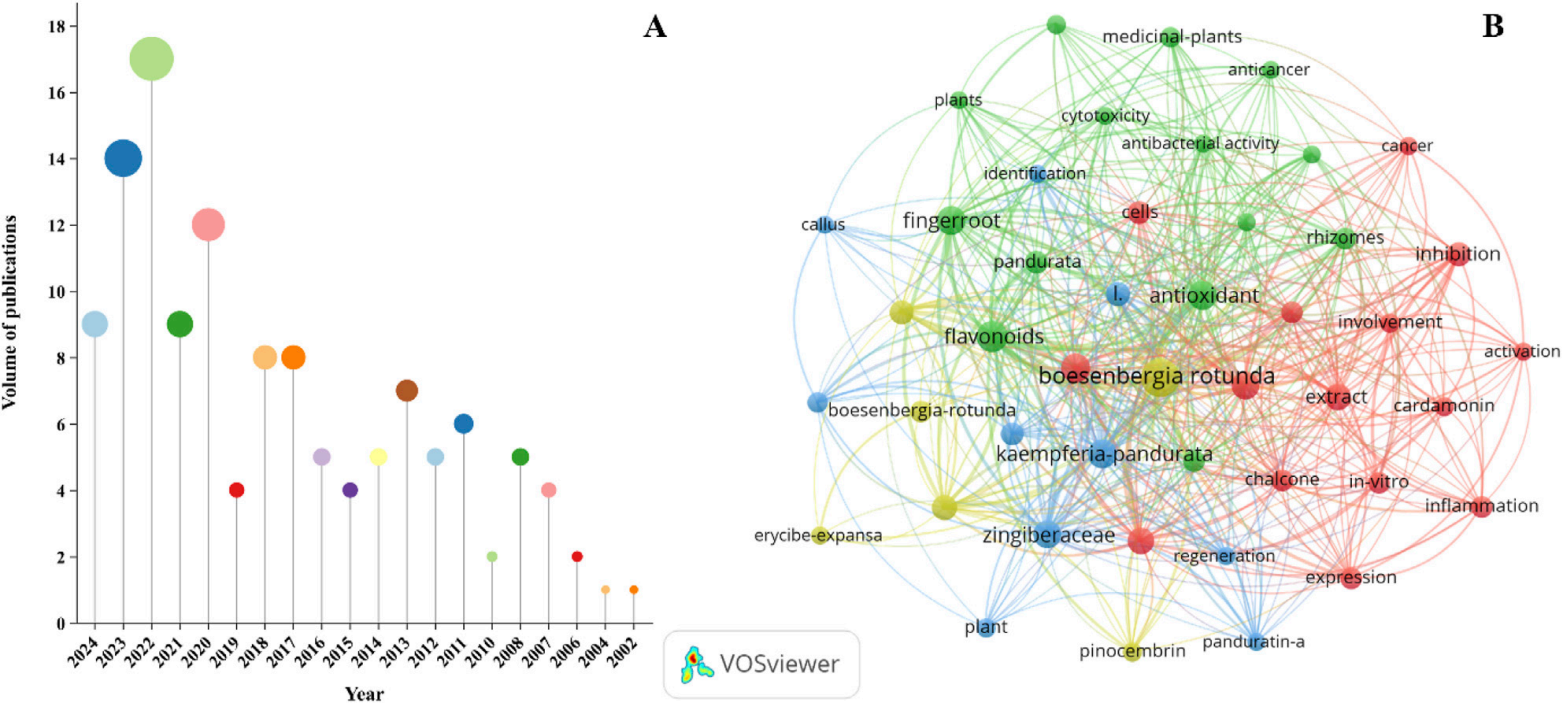
*B. rotunda* in the number of publications from 2002 to 2024 **(A)**, network visualization map of all keywords co-occurrence for *B. rotunda* **(B)**.

Bibliometric studies are instrumental in elucidating research trends, identifying hotspots, and charting developmental pathways within a delineated academic sphere, thereby providing valuable insights for strategic disciplinary planning. In this regard, we have executed a bibliometric analysis of the existing scholarly literature concerning *B. rotunda*. A comprehensive bibliometric analysis of the scientific literature on *B. rotunda* has uncovered that this species possesses a rich historical utilization in traditional medicinal practices and exhibits significant promise in modern scientific inquiry. Notably, this potential has been markedly exemplified during the preceding decade (Figure 8A), with a notable escalation in the volume of research on *B. rotunda*. This trend underscores a burgeoning surge in scholarly attention and interest towards *B. rotunda*. To enhance the comprehension of research hotspots and trends concerning *B. rotunda*, we have delineated a network visualization map encompassing all co-occurring keywords. This network diagram elucidates the pivotal themes and interconnections within the research landscape of *B. rotunda*. Flavonoids, which are the predominant bioactive metabolites in *B. rotunda*, have emerged as a focal point of investigation. These metabolites are hypothesized to possess therapeutic potential across a spectrum of diseases (Figure 8B).

In summary, there has been a marked escalation in scholarly interest towards *B. rotunda* in recent years, underscoring its potential as a focal point for contemporary scientific inquiry. It is imperative to conduct subsequent in-depth pharmacological studies to substantiate the therapeutic efficacy of flavonoid metabolites present in *B. rotunda*. Concurrently, the exploration and development of other metabolites derived from *B. rotunda* are anti-cipated to pave the way for novel drug discovery and development paradigms.

## 12 Conclusion and perspectives

The present manuscript provides an exhaustive overview of the results of scholarly research on the traditional applications, phytochemical metabolites, pharmacological properties and clinical applications of *B. rotunda*. Screening of existing studies on *B. rotunda* through various databases, screening and analysis of the literature revealed the identification and isolation of more than 200 chemical metabolites from different parts of *B. rotunda*, of which flavonoids are the most prominent bioactive metabolites. These flavonoids have significant biological activities and have been incorporated into clinical studies. In addition, the study of the multiple bioactivities of *B. rotunda* provides a solid foundation for its further development and lays the groundwork for its potential clinical applications.

While the existing body of research on *B. rotunda* has made significant strides, there are several areas that require further exploration and deepening: (1) The majority of activity studies have concentrated on specific extracts and flavonoids, with the bioactivity of other metabolites being relatively uncharted. A more exhaustive investigation into the bioactivity of *B. rotunda* is essential to maximize its clinical potential. Compile a comprehensive list of all known metabolites in *B. rotunda*, focusing on those that have not been extensively studied. Further study of the mechanism of action of key metabolites, exploration of synergistic effects with other therapeutic agents, and development of new drug delivery systems. (2) Although over 200 metabolites have been identified in *B. rotunda*, research on these metabolites remains limited. The bioactivity of monomeric metabolites has primarily focused on a select few, particularly flavonoids. Future research should aim to uncover the potential value of other metabolites and fully exploit the therapeutic potential of *B. rotunda*. (3) Quality control studies on *B. rotunda* have predominantly focused on flavonoids, with less emphasis on other metabolites. Enhanced quality control measures are needed to ensure comprehensive surveillance of all *B. rotunda* metabolites. Additionally, biological identification techniques can augment quality control efforts, and there is a need to strengthen research in this domain. Incorporate advanced analytical techniques such as LC-MS/MS and NMR to detect and quantify a broader range of metabolites in *B. rotunda* extracts. (4) There is a dearth of pharmacokinetic studies on *B. rotunda*, and further research into the pharmacokinetics of its principal active substances and extracts is imperative. Such studies are vital for informing clinical drug therapy. Start with animal models to establish pharmacokinetic parameters, followed by human studies to validate these findings and assess the pharmacokinetics in a clinical setting. Use advanced pharmacokinetic modeling and simulation tools to analyze the data and predict the behavior of *B. rotunda* metabolites in the body. (5) A comprehensive toxicological evaluation of *B. rotunda* is necessary to assess its safety. While most research has focused on *in vitro* toxicology of various extracts and metabolites, *in vivo* studies are notably scarce. Future research must expand the assessment of both *in vitro* and *in vivo* systemic toxicity and safety profiles. Conduct *in vivo* toxicological studies, including acute, subacute, and chronic toxicity tests, to evaluate the safety profile of *B. rotunda* in animal models. Expand the assessment to include organ-specific toxic effects and systemic toxicity to provide a comprehensive understanding of the safety risks associated with *B. rotunda*. (6) Ultimately, the development of *B. rotunda*-based drugs must be anchored in clinical practice. Despite its myriad biological activities, clinical research on *B. rotunda* is still limited. Therefore, prioritizing follow-up clinical studies on *B. rotunda* is crucial to ensure its effective translation into clinical applications. Identify and recruit eligible participants for the clinical trials, ensuring a diverse and representative sample population. Implement rigorous data monitoring and analysis protocols to ensure the reliability and validity of the clinical trial results, and use the findings to inform the development of effective clinical applications for *B. rotunda*. These can further improve the research on *B. rotunda* and thus promote the development of *B. rotunda* in the field of medicine and food.

Overall, *B. rotunda* occupies an important position in the field of medicine and food. Nevertheless, research on Luo Han Guo is still fragmented and incomplete, and some of its metabolites and activities have yet to be fully explored and utilized. Future research should focus on understanding the areas of pharmacology, clinical studies and pharmacokinetics. The results of this study comprehensively summarize the relevant literature and discuss the current state of research on *B. rotunda*, providing critical insights and inspiration for subsequent research efforts. These are essential to promote the comprehensive development and utilization of *B. rotunda*.

## Glossary

ALP: alkaline phosphatase
ALT: alanine aminotransferase
AMPK: AMP-activated protein kinase
AST: aspartate aminotransferase
AUC: area under the curve
Bax: bcl-associated X protein
Bcl-2: B-cell lymphoma-2
Bcl-XL: B-cell lymphoma-extra large
BNP: brain natriuretic peptide
Caspase: cysteinyl aspartate specific proteinase
CAT: catalase
C/EBPα: CCAAT/enhancer-binding protein α
CDK 4: cyclin-dependent kinase 4
CNKI: China National Knowledge Infrastructure
COX-1: cyclooxygenase-1
COX-2: cyclooxygenase-2
CK-MB: creatine kinase MB
CPT: Carnitine palmitoyl transferase
CRC: colorectal cancer
cTnI: cardiac troponin I
DPPH: 2,2-Diphenyl-1-picrylhydrazyl
DRG: dorsal root ganglion
EAAT2: excitatory amino acid transporter 2
FAS: fatty acid synthase
FBG: fasting blood glucose
FFA: free fatty acids
GFAP: glial fibrillary acidic protein
GPx: glutathione peroxidase
GSH: reduced glutathione
HDL: high-density lipoprotein
HO-1: heme oxygenase-1
HSC: hepatic stellate cells
IL-1β: interleukin-1β
IL-4: interleukin-4
IL-10: interleukin-10
IL-6: interleukin-6
IL-8: interleukin-8
iNOS: inducible NO synthase
JAK 3: janus kinase 3
KCNA 3: potassium voltage-gated channel subfamily A member 3
KIM-1: kidney injury molecule 1
LDH: lactate dehydrogenase
LDL: low-density lipoprotein
LDLC: low-density lipoprotein cholesterol
LDLR: low density lipoprotein receptor
MAPK: mitogen-activated protein kinase
MDA: malondialdehyde
MMP 12: matrix metallopeptidase 12
MMP 13: matrix metallopeptidase 13
MPO: myeloperoxidase
MRSA: methicillin resistant *Staphylococcus aureus*
NGAL: neutrophil gelatinase-associated lipocalin
NF-κB: nuclear factor-κB
NO: nitric oxide
nNOS: neuronal nitric oxide synthase
Nrf2: nuclear factor erythroid 2-related factor
PARP: poly ADP-ribose polymerase
PBG: Post-prandia blood glucose
PGE2: prostaglandin E2
PPAR-α: peroxisome proliferator activated receptors-α
PPAR-γ: peroxisome proliferator-activated receptor-γ
PKC: protein kinase C
p-Akt: phosphorylated protein kinase B
p-JNK: phosphorylated c-jun N-terminal kinase
p-p38: phosphorylated p38 mitogen-activated protein kinase
RANKL: Receptor activator of nuclear factor kappa-β ligand
RCT: reverse cholesterol transport
SOD: superoxide dismutase
SREBP-1c: sterol regulatory element binding protein-1c
STZ: streptozotocin
TAA: thioacetamide
T-AOC: total anti-oxidant capacity
TC: total cholesterol
TG: triglycerides
TGF-β1: transforming growth factor beta 1
Tim-1: T cell immunoglobulin domain and mucin domain protein-1
TNF-α: tumor necrosis factor-α
TRAP: tartrate-resistant acid phosphatase
VEGFR 2: vascular endothelial growth factor receptor 2
VEGFR 1: vascular endothelial growth factor receptor 1
VLDLC: very low-density lipoprotein cholesterol
